# Can personalized digital counseling improve consumer search for modern contraceptive methods?

**DOI:** 10.1126/sciadv.adg4420

**Published:** 2023-10-06

**Authors:** Susan Athey, Katy Bergstrom, Vitor Hadad, Julian C. Jamison, Berk Özler, Luca Parisotto, Julius Dohbit Sama

**Affiliations:** ^1^ Graduate School of Business, Stanford University, Stanford, CA 94305, USA.; ^2^ Department of Economics, Tulane University, New Orleans, LA 70118, USA.; ^3^ Amazon Lab126, Sunnyvale, CA 94089, USA.; ^4^ Business School, University of Exeter, Exeter EX44PU, UK.; ^5^ Global Priorities Institute, Oxford University, Oxford OX2 0DJ, UK.; ^6^ Development Research Group, The World Bank, Washington DC, DC 20433, USA.; ^7^ Department of Economics, Bocconi University, Milano, MI 20100, Italy.; ^8^ Department of Gynaecology-Obstetrics, University of Yaoundé I, Yaoundé, Cameroon.; ^9^ Yaoundé Gynaecology, Obstetrics and Pediatrics Hospital, Yaoundé, Cameroon.

## Abstract

This paper analyzes a randomized controlled trial of a personalized digital counseling intervention addressing informational constraints and choice architecture, cross-randomized with discounts for long-acting reversible contraceptives (LARCs), such as intrauterine devices (IUDs). The counseling intervention encourages shared decision-making (SDM) using a tablet-based app, which provides a tailored ranking of modern methods to each client according to their elicited needs and preferences. Take-up of LARCs in the status quo regime at full price was 11%, which increased to 28% with discounts. SDM roughly tripled the share of clients adopting a LARC at full price to 35%, and discounts had no incremental impact in this group. Neither intervention affected the take-up of short-acting methods, such as the pill. Consistent with theoretical models of consumer search, SDM clients discussed more methods in depth, which led to higher adoption rates for second- or lower-ranked LARCs. Our findings suggest that low-cost individualized recommendations can potentially be as effective in increasing unfamiliar technology adoption as providing large subsidies.

## Teaser

Individually tailored recommendations can be effective in increasing unfamiliar technology adoption, such as modern contraception.

## INTRODUCTION

Nearly half of all pregnancies worldwide are considered unwanted or mistimed (*1*). Delayed fertility is associated with improved maternal and child health outcomes, while unintended pregnancies are strongly predictive of short interpregnancy intervals, which are in turn positively associated with babies being born prematurely, at low birth weight, or small for their gestational age (*2*–*4*). Moderate fertility rates may also contribute to economic growth through increased female labor supply (*5*, *6*).

Modern contraceptive technology is highly effective in preventing unintended pregnancies. However, adoption in many low- and middle-income countries (LMICs) remains low: Among adult women in LMICs who report wanting to avoid a pregnancy, about a quarter of women and half of adolescent females report not using a contraceptive method (*1*). Our own formative qualitative work in Cameroon, the setting for this study, showed that there exists a multitude of demand-side (e.g., concerns over side effects, misconceptions, affordability, or opposition by male partners/spouses) and supply-side factors (e.g., lack of trained health providers and provider bias) impeding the take-up of modern methods. Increasing access to contraception by tackling some of these barriers can contribute to reductions in maternal mortality in sub-Saharan Africa, where 1 in 38 15-year-old girls will eventually die from a maternal cause (*7*). Reducing fertility uncertainty may also induce parents to increase investments in the health of their children (*8*).

We conducted a randomized experiment to tackle two of these barriers: (i) challenges in acquiring the relevant information about benefits and side effects and (ii) cost. The experiment was conducted at a women and children’s hospital in Yaoundé, Cameroon. A multidisciplinary team of health care providers, public health experts, and economists developed a tablet-based app, which assists nurses conducting contraceptive counseling sessions. During a structured discussion, the app records the clients’ fertility plans, needs, and preferences regarding contraceptive methods. The main innovation that the digital app brings to family planning (FP) counseling is an internal algorithm to rank methods according to their suitability for the client’s personal context. In the shared decision-making (SDM) treatment, the app reveals the most suitable method, and the provider suggests to the client, who makes the ultimate decision, that they discuss this method first. This contrasts with the individual decision-making (IDM) treatment, in which the app displays the methods available to the client in random order and the provider asks the client which method they would like to discuss first. SDM is thus aimed at providing the clients with information tailored to their individual needs, while IDM is meant to resemble the status quo in contraceptive counseling, in which individuals are given extensive information and then left to make their own independent choices (*9*).

The design of the two counseling interventions is firmly rooted in the debates about how to operationalize “full, free, and informed choice” that emerged from the 1994 International Conference on Population and Development (*9*–*11*). The experimental design recognizes the challenge of fully informing clients on a range of methods and, as such, is informed by a large literature analyzing the challenges individuals face in information gathering and decision-making, such as search costs, choice overload, or misinformation. Our paper relates to research establishing that organizing and prioritizing information for consumers, such as ranking the choices displayed to users, affects their decisions across a wide variety of applications (*12*–*14*), including health (*15*, *16*). It is further related to research in behavioral science as applied to decision-making contexts in health and elsewhere, in particular, the key role of choice architecture (*17*, *18*). Although we do not fully attempt to distinguish between them, there are several possibilities for how SDM could influence and potentially improve outcomes along these lines: as a simple nudge, increasing the salience of one option; as a personalized and ranked recommendation, potentially inducing reciprocity in addition to higher expected benefits from continued search for a method; or as the suggestion of a trusted professional authority. It could also encourage separate versus joint evaluation: In the behavioral science literature, it is sometimes claimed that joint evaluation of options (IDM in our case) encourages more reasoned decision-making than does separate evaluation (SDM here), and hence superior outcomes, in part because it focuses attention on relevant dimensions rather than irrelevant biases such as, e.g., gender (*19*). In our setting, however, the ranking is specifically tailored to the client’s preferences; therefore, SDM is designed to try to overcome such biases directly.

From the supply side, digital technology like the tablet-based app in our context can overcome provider bias (by recommending methods that counselors might be reluctant to discuss with certain subgroups of clients) and improve safety (by automatically eliminating methods that are contraindicated for the client). Hence, our study is also related to the literature on digital technology enhancing the capabilities of service providers, including the quality of service and compliance (*20*, *21*). In the context of health, the tablet-based app can be considered an example of medical decision support; Awaysheh, *et al.* (*22*) provides a recent comprehensive review, while Obasola *et al.* (*23*) focuses on e-health for maternal and child care in sub-Saharan Africa. By augmenting the capabilities of human workers, this type of application enables a worker to reliably provide accurate and customized information to clients.

Our study is also related to a growing literature in the US on counseling interventions—such as peer counseling, a waiting room app for contraceptive counseling, and motivational interviewing techniques allowing the client to articulate goals and discuss plans—that showed promise in increasing levels of knowledge of contraceptive effectiveness, interest in adopting the implant, and adoptions of long-acting reversible contraceptives (LARCs—i.e., the intrauterine device(IUD) and the subdermal implant) (*24*–*26*). A tablet-based decision-support tool that is similar to the one developed here but for use by women before contraceptive counseling was found to be acceptable to women in a formative evaluation (*27*), with a cluster-randomized trial finding no effects on contraceptive continuation after 7 months but improved contraceptive knowledge, informed decision making, and enhanced client experiences (*28*).

We cross-randomized a discount treatment, in which the app revealed randomly assigned discounts for modern contraceptive methods. The discount treatment relaxes the liquidity or credit constraints that prevent clients from adopting LARCs due to their high upfront costs, although they typically have lower per-month costs of protection from unintended pregnancies compared with short-acting methods, such as the pill or the injectable (Depo-Provera). Providing vouchers that fully or partially subsidize FP services increases the use of modern contraceptives among women—especially among young, unmarried, or low-income women (*29*–*35*). However, subsidies may not always be effective when there are multiple barriers to access. Shah *et al.* (*36*) find that free provision of modern contraceptives to adolescent females at girls’ clubs in Tanzania did not lead to increased take-up. Ashraf *et al.* (*29*) find that the provision of vouchers to married women in Zambia in the presence of their husbands reduces their redemption compared to them being offered when the women are alone.

## MATERIALS AND METHODS

### Study setting and target population

The study was conducted at the Hôpital Gynéco-Obstétrique et Pédiatrique de Yaoundé (HGOPY), a women’s hospital in the capital of Cameroon. In preparation for an adaptive experiment aiming to reduce unintended pregnancies among its clients, the study team organized a pilot in December 2019. This phase served to introduce the app to nurse counselors; provide FP training to nurses in other units, such as maternity and gynecology; iron out the study protocols; and, perhaps, get all trained nurses to become comfortable with providing FP counseling under either approach—IDM and SDM—using the app. Despite the declaration of the global pandemic by the World Health Organization in March 2020, which reduced the number of clients presenting at HGOPY, these goals were successfully achieved by 9 June 2020. However, partly because of the disruptions caused by the pandemic, the study team was not ready to launch the adaptive experiment at that time. Instead, the team decided to run a static experiment with fixed and equal probabilities of assignment to each of the two interventions that were cross-randomized: counseling and discounts, both of which are discussed in detail later in this section. This experiment ran for 9 months until the launch of the adaptive experiment on 9 March 2021, to which we refer as the study period for the remainder of this paper, and provides the basis for this study.

Women aged 15 to 49 who received FP counseling at HGOPY by a trained provider using the app during the study period were included in the analysis. We excluded clients who (i) wanted to become pregnant within the next 12 months—about 5% of the all clients, (ii) were pregnant at the time of their consultation and had not yet come back to give birth at HGOPY by the end of the study period—as we could not yet observe their outcomes, and (iii) completed their consultation without being exposed to either intervention—this included primarily women who wanted to discuss side effects of their current method and who continued using it without needing to renew it. During the study period, 1008 clients were counseled at HGOPY, 784 of whom are in the study. Approximately half of these clients (57%) presented at the FP unit—either seeking counseling to simply receive information; adopt a new method; switch to another method; or renew, manage the side effects of, or discontinue their current method. The remaining clients mainly presented at the maternity and gynecology wards: some had just given birth; some were pregnant and receiving antenatal services; some might have returned to the hospital postpartum for a check-up or for their infants to receive vaccinations; yet others might have presented with a gynecological problem.

Table 1 shows the characteristics of the study sample (columns 1 to 3) and those of a comparable sample of women from the Yaoundé stratum of the Demographic and Health Survey (DHS) of Cameroon in 2018 (column 4). The average client recruited in our study is 29 years old, more than 2 years older than that in the DHS sample. The study sample is more or less equally divided between women who are single, cohabiting with a partner, and married. Approximately a quarter of them are students. HGOPY clients in the study sample are twice as likely to have some tertiary education as the DHS sample and more likely to be salaried employees. However, they are also more likely to have only primary (or lower secondary) education—perhaps due to the service social provided by HGOPY for women with lower socioeconomic status, reflecting the broad range of clients who frequent HGOPY. The average number of children is 2.75, and 25% of clients report wanting no more children. As HGOPY clients in the study sample are older, on average, than the DHS sample in Yaoundé, they are also more likely to be married, have more children, and want to wait longer before becoming pregnant. They are also much more likely to have given birth in the past 3 months (56% versus 5%). As discussed in the “Contraceptive methods” section below, we refer to the IUD and the implant as LARCs and the pill and the injectable (Depo-Provera) as SARCs (short-acting reversible contraceptives) throughout our study. Few clients were using a LARC or a SARC in either sample.

**Table 1.** Client characteristics. This table shows client characteristics for everyone and by department in the study sample and in the Demographic and Health Survey (DHS) of Cameroon in 2018; column 1 includes the full sample of clients in the study sample; columns 2 includes clients who received a consultation at the family planning (FP) department, i.e., who visited the FP unit through their own personal initiative; column 3 includes clients who visited another hospital department, primarily maternity and gynecology; and column 4 includes only individuals from the Yaoundé stratum of the DHS. MiM refers to Method in Mind clients, who are individuals that visited the hospital seeking a specific method, which they either wanted to adopt or renew; this category is not present in the DHS data. Adolescent refers to individuals between ages 15 and 19. In the DHS, data on the body mass index (BMI) is only collected for mothers of children born in the 3/5 years preceding the survey (months 0 to 59 before the survey) for a total of 467 respondents. Both DHS and the study sample are restricted to women ages 15 to 49, who are not pregnant, and do not want to become pregnant within the next 12 months.

	(1)	(2)	(3)	(4)
	Study sample			DHS sample
	Full sample	FP	Mat./Gyn.	Yaoundé
	Mean/(SD)	Mean/(SD)	Mean/(SD)	Mean/(SD)
Age	29.27 (7.03)	29.64 (7.05)	28.78 (6.98)	26.98 (9.05)
Adolescent	0.09	0.07	0.10	0.28
BMI	27.53 (4.86)	27.45 (4.97)	27.65 (4.71)	25.09 (3.76)
Unmarried couple cohabiting	0.37	0.33	0.41	0.16
Married	0.33	0.39	0.26	0.23
Education: Tertiary	0.42	0.47	0.35	0.21
Education: Secondary	0.23	0.22	0.24	0.64
Education: Primary/Lower sec.	0.33	0.29	0.38	0.13
Salaried employee	0.32	0.34	0.28	0.18
Self-employed	0.21	0.19	0.24	0.29
Student or apprentice	0.23	0.22	0.24	0.31
Pregnancies, total	3.69 (2.33)	3.65 (2.32)	3.74 (2.35)	1.86 (2.03)
Children alive today	2.75 (1.81)	2.73 (1.80)	2.78 (1.83)	1.75 (1.86)
Ever gave birth (live or still)	0.94	0.94	0.94	0.63
Gave birth ≤3 months	0.56	0.34	0.84	0.05
Wants no more children	0.25	0.24	0.27	0.28
Wait 1 to 3 years before next pregnancy	0.36	0.36	0.36	0.16
Wait >3 years before next pregnancy	0.39	0.40	0.37	0.15
Currently using a LARC	0.03	0.05	0.01	0.05
Currently using a SARC	0.04	0.05	0.02	0.06
Currently using other method	0.05	0.08	0.01	0.24
MiM	0.49	0.56	0.39	–
*N*	784	448	336	1067

### Interventions

The interventions are centered around the use of a tablet-based app, developed as a job-support tool for nurse counselors providing FP services (see Supplemental Text section D for a detailed description of the app and its main features). All nurse counselors at HGOPY were given state-of-the art FP training, designed by professors of obstetrics and gynecology and other reproductive health experts in Cameroon, before conducting counseling sessions with HGOPY clients using the app.

Formative qualitative work in Cameroon before the development of the app and the design of the trial suggested that concern and confusion about side effects—both among clients and providers—were some of the main barriers to the take-up of modern contraceptives. Discussion of possible side effects during counseling may be particularly important: For example, Hubacher *et al.* (*37*) provides supporting evidence from a trial in the US that the main reported reason for discontinuation of modern contraceptive methods is side effects, with this being especially strong for users of LARCs. As such, the app combines the extant literature on the expected side effects of each method with the client’s preferences on how much they care about avoiding side effects, elicited during counseling, and ranks the methods for each client according to its suitability. Side effects are only one of the criteria used by the app to provide individually tailored rankings: Supplemental Text section D.3 provides a more detailed description of the criteria the app uses to rank contraceptive methods.

Our formative qualitative work also suggested that many providers were reluctant to discuss certain methods, especially long-acting ones, with certain subgroups of clients, such as adolescent, unmarried, or nulliparous clients. Hence, the job-support tool aimed to reduce provider bias by structuring the discussion around a given ranking of methods. As the app records all actions with time stamps, it is difficult for counselors to deviate from the structure of the counseling session in its algorithm, as metadata and paradata (regularly uploaded to the cloud from each session) would quickly identify providers with unusual activity. In both its purpose as a tool that accommodates shared decision-making by providing individually tailored recommendations based on elicited client goals and preferences and as a tool that can enhance a better discussion by empowering the client, the app is not dissimilar to the My Birth Control app, designed for use by clients before contraceptive counseling (*27*).

For the purposes of our study, the app served as both the platform through which the counseling approach and prices of contraceptive methods were randomly assigned to each client and the data collection tool to assess the impacts of these interventions. Every client enrolled in the study was counseled by a trained provider using the app.

#### Counseling approach

The structure of the counseling session guided by the app is not fundamentally different than the accepted best practices used around the world. The main innovation it brings to FP counseling is a small but important paradigm shift with respect to shared decision-making: The app uses an algorithm to internally rank methods from most to least suitable for each client using her elicited goals and preferences. In the established, or status quo, approach to counseling, these tailored rankings are not revealed to the client (nor to the provider): Instead, the client is given information about all contraceptive methods and asked to choose the method she would like to discuss. The counselor is expected to provide no guidance or advice during this IDM process. In our alternative approach, the app encourages SDM by revealing the top-ranked method for the client according to the app’s internal algorithm with the nurse counselor proposing to discuss that method first. To test the effectiveness of this approach against the status quo, clients were randomly assigned to IDM or SDM with equal probability:

1) IDM: The tablet displays all available modern contraceptive methods that have not been ruled out by the client or contraindicated due to medical eligibility. The provider gives basic information on each available method (in order of the methods displayed on the tablet screen, which is randomized) covering its use, duration, and typical use effectiveness. These quick descriptions are designed to take 1 to 2 min per method or 5 to 6 min overall. The counselor then asks the client which method she would like to discuss and provides detailed information with the help of the relevant cue card. The client can then either choose this method or discuss another one (of her choice from the same unranked list). This process is repeated until a decision is made. The app’s tailored ranking of methods is never revealed (the client does not know that there is an internal ranking).

2) SDM: The tablet only displays the most suitable (top-ranked) method for the client according to the ranking algorithm of the app. The provider tells the client that “… while there are a number of suitable methods for her, based on their discussion, the displayed method is most suitable for her needs,” and asks her if she would like to discuss this method first. If the client answers “no,” then the next highest ranked method is displayed, and the process is repeated until the client decides to discuss the recommended method (or decline all of them). When the client answers “yes,” the provider uses the appropriate cue card to describe the method in detail. The client can then decide whether to choose this method (with an intent to adopt it) or discuss the next method recommended by the app. Again, this process is repeated until a decision is made.

The randomization occurs toward the end of the consultation when the clients are ready to choose a method, after the discussion necessary for the client to make an informed choice and for the app’s internal algorithm to rank the methods has taken place, but before the random discounts are revealed. The counseling experiment aims to isolate the effect of informing the client that the app ranks all available methods according to their suitability for her needs, proposing to discuss them in this order of the rankings starting from the top, and revealing the methods one by one—rather than asking her to decide which method she would like to discuss from a list of unranked options.

**Fig. 1.**
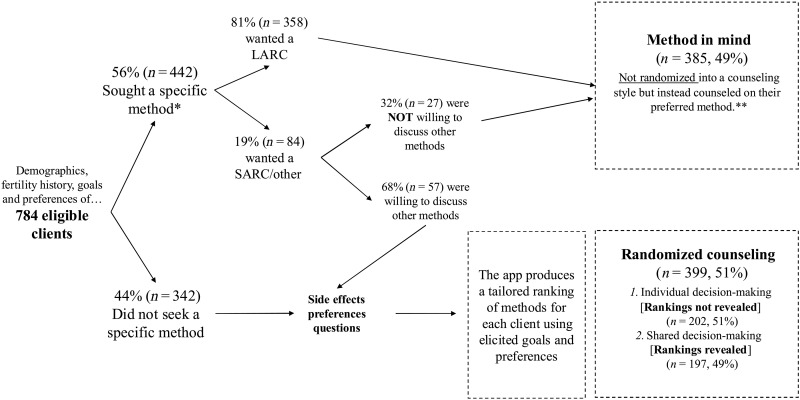
FP counseling flowchart. *Or wanted to renew their current method without discussing other methods (approximately 10% of this group of clients). **In rare cases where a client’s preferred method is contra-indicated they were counselled on the next most suitable method according to a default ranking.

However, not everyone was randomly assigned to a counseling condition: slightly over half of the eligible clients visited the hospital seeking a specific method, which they either wanted to adopt or renew. The pilot phase preceding the study period had clearly revealed that such clients did not want, or need, to sit through a time-consuming session, where either all methods were summarized to them (IDM) or some discussed in detail (SDM). Figure 1 shows a flowchart of the paths taken by first-time clients through the app. It is important to note that these clients were expressing a strong intention to adopt a specific method: The question in the app reads “is there a specific method that you absolutely want to adopt?”. When clients expressed their intentions, regardless of the method sought, the counselor would engage them in a discussion to try to understand the source of their interest and their level of knowledge—both of the method sought and of contraception more broadly. If the nurse counselors, who were trained extensively, concluded that the client’s intentions were not sufficiently strong, then they would suggest the possibility of discussing other methods. If the client refused the providers’ suggestion, then they would proceed as described below.

For clients seeking to adopt a specific method, the app follows a slightly different protocol depending on whether that method is a LARC or not. Clients who want to adopt or renew a LARC are directed to the method choice section to discuss their desired method in detail, after a check on their medical history to assess any contraindications: Approximately 80% of clients seeking to adopt a specific method fall into this category. In contrast, for the remaining 20% who want to adopt or renew a SARC (or other methods with lower typical use effectiveness), the app prompts the provider to inquire whether the client would be willing to discuss other methods that are more effective in preventing pregnancies and might be more suitable for her. Such clients (68%) were willing to consider other methods and thus eligible to be randomly assigned to a counseling intervention, while the remaining clients were directed to the method choice section to discuss their preferred (non-LARC) method in detail. This resulted in roughly half of the clients being randomly assigned to a counseling intervention, while the other half were counseled on their preferred method (Fig. 1). We refer to the latter group as having a method in mind or MiM for short. MiM clients are not directly offered the method they want: Rather, in addition to an assessment of contraindications, they are fully counseled on their desired method with the aid of a cue card to ensure that it is suitable for their needs and preferences. In cases where an MiM client wanted to discuss other methods after their preferred method, which happened less than 5% of the time, the nurses suggested another method according to a default ranking (by typical use effectiveness) provided by the app for MiM clients.

There are two motivations for the asymmetry in how the app treats MiM clients seeking a LARC versus a SARC. First, LARCs have much higher typical use effectiveness in preventing unintended pregnancies, which is an important attribute of contraceptive methods. Second, knowledge of LARCs is typically lower than that of SARCs: The 2011 DHS shows if a woman had heard of a LARC, she almost certainly had also heard of a SARC. However, the reverse is not true: Among clients who had heard of a SARC, only two thirds had heard of a LARC. This makes the odds of knowing about the other group of contraceptives 25 times higher among people who heard of a LARC versus a SARC. (While these odds have decreased considerably to approximately 7 in the 2018 Cameroon DHS, which was not yet publicly available during the study preparations, the information asymmetry still exists between SARCs and LARCs among women of reproductive age in Cameroon). As Senderowicz (*10*) notes, while it may not be possible to be informed about all possible methods, it is often desirable to be informed about more than two of them (preferably from different groups, such as long- versus short-acting). Data from Cameroon suggest that if someone knows of a LARC, then they are almost guaranteed to be familiar with at least two methods—one from each group (long- versus short-acting). This is not the case for clients seeking a SARC: They might have never heard of the implant or the IUD. Hence, the app nudges MiM clients seeking a SARC toward the possibility of discussing other methods first via random assignment to IDM or SDM.

#### Discounts for modern contraceptive methods

Eligible clients who received a FP consultation by a trained provider using the app were offered randomly varying discounts for modern contraceptives. Contraceptive prices and the counseling style are fixed for each client for 1 year per the random allocation at their initial visit, meaning that return clients face the same set of prices if they want to adopt a new method or switch their current one. The four modern contraceptive methods available at the hospital were grouped into two categories: LARCs (i.e., the copper IUD and the implant) or SARCs (i.e., the pill and the injectable). LARCs were offered at five different price levels with equal probability, which we collapse into two bins for statistical power: full price [Central African Francs (CFA) 4000] and discounted price (CFA 2000; 1000; 150; and free). Removals of LARCs are free for all study participants whenever they wish to switch or discontinue their method. SARC prices were cross-randomized at two price levels with equal probability: Full price (CFA 1250 for the injectable and 1500 for the pill) or free. Both short-acting methods need to be renewed every 3 months, and the clients could do so at the same price offered at the initial consultation, which was valid for a period of 1 year, representing three renewals. Note that because of the very low take-up of SARCs among the study population and the lack of impact of offering discounts for SARCs, the study team discontinued offering random discounts for SARCs on 20 January 2021, meaning that they were offered at their full price for the remainder of the study period. For this reason, we do not focus on SARC discounts in the analysis.

Two details about the implementation of random discounts might matter. First, the prices for all modern methods were revealed at once and after the client had chosen a method they wanted to adopt unless they inquired about prices earlier on in the counseling session, which happened only 4% of the time. This decision was made after careful deliberation with health care providers at HGOPY: The study team wanted to avoid price discounts possibly skewing the discussion of suitable methods for the clients. Second, the reader might worry about the clients (alone or in collaboration with the providers) trying to “game” the system to receive higher discounts—for example, by asking the counselor to start a new app session or by returning later. Carefully developed study protocols for return clients, monitoring the data from the tablets (including meta- and paradata), and monthly audits of HGOPY’s own administrative data allow us to rule out the possibility of clients redrawing a new set of prices.

We can also largely rule out any meaningful bias in our impact estimates due to clients visiting nearby FP clinics to receive the same services at lower prices than those they were offered at HGOPY: First, even the highest LARC prices in our study constitute a nonnegligible discount over the regular prices offered at HGOPY and at other publicly funded hospitals and clinics in Yaoundé. Second, we surveyed nearby private and public-private clinics to get price estimates for contraceptive methods and associated services, which exceeded the costs borne by clients in our study (see table S5). Third, the randomized price of adopting each contraceptive method in our study covers all associated services, which includes FP counseling, removals of LARCs, follow-up services to address side effects and complications, and free provision of condoms. Table S5 shows that separate charges for LARC removals and FP counseling in other facilities can be quite expensive. Last, follow-up surveys we conducted with a later sample of clients, who were recruited during the adaptive experiment, indicate that only a negligible share of HGOPY clients sought FP services elsewhere following their consultation at HGOPY, less than 2.5%.

### Contraceptive methods

The methods displayed by the app are the copper IUD, the implant, the injectable, the pill (combined or progestin-only), and lactational amenorrhea method (LAM) if the client is eligible. The Levonorgestrel (LNg) IUD was not available at HGOPY during the study period. Female sterilization in Cameroon is rare, subject to (age- and parity-based) eligibility rules, and often requires layers of approvals, including the consent of the husband. Condoms (male and female) are available at HGOPY, and every counseling session ends with a discussion of dual protection against sexually transmitted infections and free provision of condoms. Each nurse counselor has a set of cue cards for all the methods mentioned above, plus male sterilization, standard days method, and emergency contraceptive pills. If a client discusses (or skips) all the methods listed in the app but chooses to adopt none of them, then the nurse counselor presents the client with a summary cue card that shows these other methods that the clients can adopt to continue the discussion. Please see Supplemental Text section D.4 for an example of the cue cards used by nurse counselors at HGOPY.

In this study, we deviate from a definition of short-acting methods that might typically include the pill, condoms, LAM, the standard days method, and emergency contraception and refer to only the pill and the injectable as SARCs. We do not categorize the injectable as a LARC because of the large differences in typical use effectiveness: 0.05% of women using the implant experienced an unintended pregnancy within the first year of use, the same figures were 0.8% for the copper IUD, 6% for the injectable, 9% for the pill, and 18% for the male condom. LAM, when used correctly, is as effective as a short-acting method for up to 6 months after giving birth (*38*). Combined with the fact that the injectable needs to be renewed every 3 months, we felt that categorizing it as a SARC was reasonable. Furthermore, we did not include short-acting methods other than the pill and the injectable in our definition of SARCs, because these are the only short-acting methods that are always ranked by the app for all clients and for which we experimented with discounts. In the “Main results” section, we show that our conclusions do not change if we define SARCs to include all methods other than LARCs.

### Data

The app collects a rich set of client characteristics, including demographics, weight, and blood pressure, as well as relevant medical and birth history. It also records the client’s fertility preferences, prior experience with contraception, whether and why they seek to adopt a specific method, their preferences regarding side effects, and which method—if any—they adopted at the end of the consultation. For each method discussed during the counseling session and not chosen by the client, the app records the reason why she did not want to adopt it.

We supplement these data with follow-up surveys of clients who were enrolled into an adaptive experiment that followed the study period; we will henceforth refer to this sample as the follow-up cohort. These data, collected via phone surveys, include information on quality of care during and client satisfaction with the FP counseling sessions (2-week follow-up surveys), as well as rates of method continuation and satisfaction (16-week follow-up surveys) and provide suggestive evidence on whether the method choices the clients made and the outcomes they experienced as a result of those decisions were aligned with their preferences. The follow-up cohort was counseled between the 19th of January 2021 and the 29th of June 2022, which partly overlaps with the present study period. Table S1 shows that this sample shares the same characteristics as our study sample and is similarly different than the random sample of women in the 2018 Cameroon DHS. These clients were assigned to exactly the same interventions as clients in the study sample—only the assignment probabilities to each intervention arm differed under the adaptive experiment phase.

### Outcomes

The primary outcomes considered in this study are the shares of clients who adopted (i) a LARC, (ii) a SARC, or (iii) neither. We denote the last group as having adopted neither although it includes clients who adopted LAM, condoms, or other traditional methods. We mainly focus our attention on the adoption of LARCs due to low demand for SARCs among our study population (table S2 provides a detailed breakdown of the method mix pre- and postcounseling). The primary outcomes are constructed using data collected on the tablets, which are cross-checked with hospital administrative records that are further verified by a third-party independent auditor. A nonnegligible number of clients do not adopt a method during their initial visit but return to HGOPY to adopt a method later—e.g., after taking some time to think about their decision; having discussed it with their partners, spouses, or parents; needing to collect the money necessary to adopt the method; etc. For these clients, the data across consultations are linked so that the most up-to-date outcome is considered. Similarly, some clients may return to the hospital to switch or discontinue their adopted method, which are reflected in the primary outcomes if they happen within the study period. Given that approximately 90% of clients who returned to HGOPY to adopt (or remove or switch) their chosen method have done so within 100 days of their initial visit, we track outcomes for clients whose initial visit was between 9 June 2020 and 9 March 2021, who may have returned to HGOPY for a follow-up visit until 17 June 2021. In the “Mechanism and heterogeneity of impacts” section, we also examine secondary outcomes, which may help to shed light on mechanisms of intervention impacts. These include the number of methods discussed in detail, as well as the personalized rankings of the methods discussed (or adopted) per the app’s algorithm. Last, we examine outcomes related to contraceptive concordance, which were described in the previous subsection.

### Ethical considerations

The study protocols were approved by Cameroon’s National Ethics Committee for Human Subjects Research, the National d’Ethique de la Recherche pour la Humaine (CNERSH; decision no. 2019/08/1183/CE/CNERSH/SP). The study also received administrative authorization from the Ministry of Health’s [Min Division of Health Operations Research (DROS; decision no. D30- 760/L/MINSANTE/SG/DROS)]. Last, the protocols were also approved by our own institutional review board (decision no. 780/CIERSH/DM/2018). The adaptive experiment, which followed this study, is registered at the American Economic Association's registry for randomized trials and can be accessed at the following link: https://www.socialscienceregistry.org/trials/3514. Study protocols submitted for ethics review are included in the registration, which cover the full set of study procedures, including, but not limited to, data management and information security, enrollment criteria, consent procedures, and treatment of adverse reactions.

## RESULTS

### Main results

**Fig. 2.**
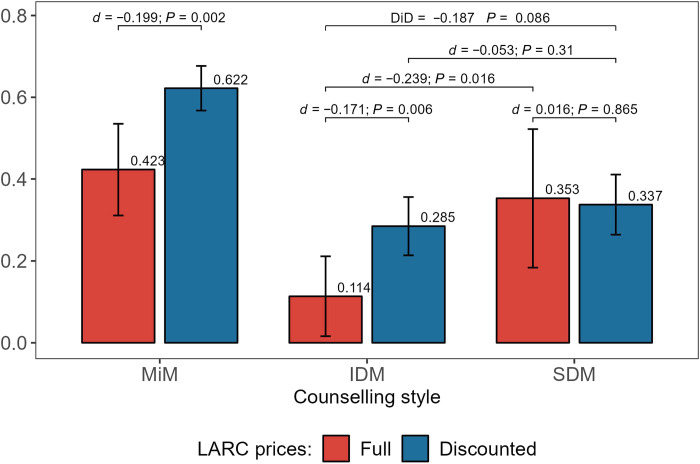
Fraction of clients who adopted a long-acting reversible contraceptives (LARC). The figure shows the fraction of clients who adopted a LARC during the study period under Discounted and Full LARC prices across counseling styles. The lines above the bars show the estimate (d) and *P* value (*P*) from a *t*-test of the difference in means between the two indicated groups. The bar labeled DiD indicates the difference-in-differences estimate between the two randomized counseling interventions (IDM-SDM) and LARC price discounts (Full-Discounted).

Tables S3 and S4 show that client characteristics are balanced across randomly assigned LARC prices and counseling interventions, respectively. As we use administrative data from the tablets for our main analysis, our outcomes are not subject to attrition. Figure 2 presents the main findings from our experiment. It shows the share of clients who adopted a LARC by the three counseling regimes (MiM and randomized into IDM or SDM), each of which is further disaggregated by whether LARCs were offered at a discounted or full price. Figure S1 shows the same results with discounts grouped into free, discounted, and full prices. The results are similar to the main specification.

Among clients with a MiM, 42% adopted a LARC at full price. Offering discounts for LARCs increased this share by 20 percentage points (pp). MiM clients differ from other study participants: They are older, more likely to be married, have higher levels of education, and want no more children, all of which may help explain their higher demand for LARCs.

Among those who were unsure about the method they would like to adopt, when clients were randomly assigned to IDM, only 11.4% of them adopted a LARC at full price. Receiving a discount, on average, increased this share to 28.5%. Under SDM, the uptake of LARCs at full price jumped to 35.3%—an increase of more than 300% over IDM. SDM seems to have also caused clients to become less sensitive to LARC prices: Offering them discounts made no difference to LARC adoptions. The difference in the effect of providing discounts for LARCs between IDM and SDM is 18.7 pp (*P* = 0.086). Neither the provision of discounts nor the counseling regime made a significant difference in the uptake of SARCs, which was low in our study population (fig. S2). In particular, the large gains obtained under SDM in the uptake of LARCs at full price is accompanied by a similarly large reduction in the share of clients adopting no method and no change in the share of clients adopting another method. Figure S3 shows treatment effects using a broader definition of SARCs that also includes condoms, LAM, standard days method, and emergency contraception.

As LARC uptake is significantly higher under SDM than IDM, it follows that the probability of unintended pregnancy is lower among SDM clients. While prevention of unintended pregnancies is clearly an important outcome for FP interventions, it is not the only one. It is also critical that the outcomes (including the decision to adopt no method) are aligned with client preferences, that the quality of care is high, and that the clients are satisfied with the services they receive. Hence, before examining the mechanisms through which SDM increased the uptake of LARCs, we present some evidence on these outcomes.

Table S8, using survey data collected from the follow-up cohort mentioned in the “Data” section, shows that the quality of care received by the clients was high (panel A). The quality of care index is based on a measure developed and validated by Jain *et al.* (*39*) and includes information on quality domains such as method selection (whether the counselor asked questions that are pertinent for selecting a suitable method), effective use (informed about the possibility and management of side effects), and continuity of care (informed about the possibility of switching to another method). Values of the quality sub-indices are high and not differential across SDM and IDM (the “Audio and Visual Privacy” sub-index is the only exception because physical space for counseling and administration of FP services is limited at HGOPY). Client satisfaction with FP services in general and the counseling session in particular were equally high in both groups, and more than 90% of all clients reported that they were likely to return to HGOPY for FP services (panel B).

Another indication of the concordance of clients’ choices with their preferences is whether they continue to use the methods they adopted: If the increased LARC adoptions among SDM clients are, on average, not aligned with their preferences, then we should observe more discontinuations or switching to other methods. To examine this issue, we can track the decisions clients in our study made over time—from the day of their initial counseling until several months later. Figure S4 replicates our main impact findings presented in Fig. 2 above using adoption decisions made on the day of the first counseling session for each client and shows that the main impacts are robust to defining adoptions in this way. Comparing these two figures, we can see that LARC adoptions increase over time across all groups but that the gap between IDM and SDM increases slightly. Figure S5 confirms this pattern of increasing uptake over time for both full-priced and discounted LARCs, with the gap in cumulative adoptions between IDM and SDM widening slightly over time. Last, table S7 shows (using data from 16-week follow-up surveys with the same follow-up cohort used in table S8) that later adoptions of LARCs by clients who adopted neither a LARC nor a SARC on the day of their initial counseling far exceed discontinuations (or switching methods) by those that initially adopted a LARC, consistent with fig. S5, which shows the cumulative LARC adoptions net of discontinuations and switching in our study sample.

The evidence presented so far suggests that the SDM approach in counseling led to an increase in the take-up of LARCs. Furthermore, the quality of FP services and client satisfaction with those services are high overall and no lower in SDM than IDM. Similarly, discontinuation rates for LARCs are low—the number of LARC users increases over time net of discontinuations and switching. Taken as a whole, these findings suggest that SDM not only lowered the probability of unintended pregnancies but it also resulted in outcomes that are highly likely to be aligned with client preferences. We now turn to an investigation of mechanisms through which these effects were obtained.

### Mechanism and heterogeneity of impacts

In Fig. 2, we have seen that counseling with SDM more than triples LARC adoptions at full price. In this subsection, we explore one potential explanation behind this effect, namely, an increase in the number of modern contraceptive methods discussed in detail by the clients under SDM, as might be predicted by a consumer search model. As a brief reminder, in either counseling regime, once a detailed discussion of a particular method is completed, the client is asked whether she would like to adopt this method or discuss another one. This continues until the client chooses a method (87% of clients choose a method) or declines to discuss any more methods (13% of clients choose no method). We make a distinction between the client choosing a method and adopting one because many clients choose a method but leave the hospital without adopting one.

**Fig. 3.**
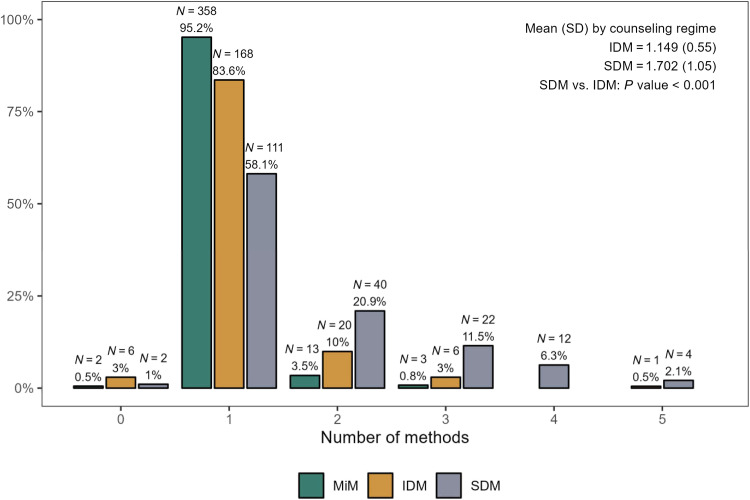
Number of methods discussed in detail. This figure shows the number of methods discussed in detail under each counselling style. The *P* value in the top right corner refers to a *t*-test of the equality of means in the individual decision-making (IDM) and shared decision-making (SDM) groups.

Figure 3 shows the distribution of the number of methods that are discussed in detail by counseling regime. As expected, 95% of MiM clients discussed only one method before choosing a method. However, an overwhelming majority, 84%, of IDM clients also discussed only one method in detail. In contrast, more than 40% of SDM clients discussed at least two methods and 20% discussed three or more methods in detail. Overall, SDM clients discussed 0.55 more methods in detail, almost a 50% increase over a mean of 1.15 in IDM (*P* value < 0.001). The finding that clients invest in discussing more alternatives under SDM is consistent with the theoretical search model of Athey and Ellison (*12*), in which consumers have heterogeneous costs of evaluating alternatives to find a product that meets their individual needs and compare the expected benefits of searching to search costs. In this model, lists sorted by expected match quality, much like the method rankings revealed in SDM in our study, increase both the number of products evaluated and consumer surplus as compared to randomly sorted lists. In other words, because the personalized rankings generated by the app were never revealed to them, IDM clients stopped their search for a method that meets their needs much faster than SDM clients, who mostly followed a predictable top-down search strategy using their ranked list—discussing the second-ranked method if they did not like the first, the third-ranked method if they did not like the first two, and so on (fig. S6). The higher number of methods discussed by SDM clients, combined with the fact that they were not less likely to discuss any given method (fig. S7), also ameliorates the potential concern that providing tailored recommendations to clients might undermine the informational value of FP counseling sessions—by causing them to consider and learn about a smaller number (or subset) of methods.

As the rankings provided by the algorithm are based on the pre-intervention characteristics and elicited preferences of the clients, our experimental design enables straightforward inference about heterogeneous treatment effects (HTE). This analysis allows us to examine how often IDM clients, who were unaware of the rankings, discussed and adopted the methods that were deemed most suitable for them. Furthermore, identifying the subgroups for which these outcomes improved among SDM clients allows us to speculate about potential pathways of impact.

Table 2 presents HTE for number of methods discussed and LARC adoptions by the personalized rankings of methods generated by the app for each client, which were revealed to both the nurse counselor and the client in SDM, but to neither in IDM. Note that heterogeneity analysis by the internal app rankings is equivalent to examining HTE by clients’ answers to questions that are used to determine the rankings (see Supplemental Text section D.3), which have been aggregated into a personalized score for each method. Clients with the same rankings in both the IDM and SDM have similar preferences over side effects and how long they would like to wait before becoming pregnant. This analysis is restricted to clients who were randomized into IDM or SDM (Fig. 1) and is averaged over all randomized prices. We omit MiM here because they are a select group in which only the discount experiment was carried out. We also average over all randomized prices because the sample size at any given price would be too small for any meaningful analysis of heterogeneity.

**Table 2.** Heterogeneity of treatment effects by the internal ranking of methods. SEs in parentheses. The injectable was never the top-ranked nor the second-ranked method. For the purpose of grouping the two methods commonly ranked at the top, short-acting reversible contraceptives (SARC) includes pill and lactational amenorrhea method (LAM) in this table, while other includes the following combinations: pill-intrauterine device (IUD), pill-Implant, LAM-IUD, and LAM-pill. Individual decision-making (IDM) row represents the mean value of the outcome for the IDM group. Shared decision-making (SDM) row represents the mean value of the outcome for the SDM group. SDM-IDM is the differences of mean between SDM and IDM. While there are 399 clients who were randomized into IDM or SDM in our analysis, we have incomplete information on the discussion of methods for seven clients, leaving us a sample size of 392 for the analysis required for this table.

	(1)	(2)	(3)	(4)	(5)	(6)
	**First-ranked method–second-ranked method**					**All**
	**IUD-implant**	**Implant-IUD**	**IUD-SARC**	**Implant-SARC**	**Other**	
**Panel A. Number of methods discussed**						
IDM	1.033	1.071	1.175	1.313	1.414	1.149
SDM	1.788	1.318	1.845	1.643	1.913	1.702
SDM-IDM	0.755	0.247	0.670	0.330	0.499	0.552
	(0.133)	(0.128)	(0.207)	(0.299)	(0.270)	(0.084)
**Panel B. Discussed top-ranked method**						
IDM	0.300	0.661	0.425	0.500	0.241	0.433
SDM	0.827	0.909	0.810	0.929	0.739	0.838
SDM-IDM	0.527	0.248	0.385	0.429	0.498	0.405
	(0.081)	(0.082)	(0.091)	(0.154)	(0.123)	(0.044)
**Panel C. Discussed second-ranked method**						
IDM	0.483	0.179	0.300	0.250	0.345	0.323
SDM	0.654	0.182	0.379	0.286	0.391	0.403
SDM-IDM	0.171	0.003	0.079	0.036	0.046	0.080
	(0.093)	(0.078)	(0.099)	(0.167)	(0.137)	(0.049)
**Panel D. Discussed third- or lower-ranked method**						
IDM	0.250	0.214	0.425	0.563	0.655	0.358
SDM	0.192	0.159	0.500	0.357	0.609	0.340
SDM-IDM	−0.058	−0.055	0.075	−0.205	−0.046	−0.018
	(0.079)	(0.080)	(0.103)	(0.185)	(0.137)	(0.048)
**Panel E. Adopted a LARC**						
IDM	0.283	0.357	0.200	0.063	0.138	0.249
SDM	0.365	0.455	0.259	0.357	0.304	0.346
SDM-IDM	0.082	0.097	0.059	0.295	0.166	0.097
	(0.089)	(0.099)	(0.088)	(0.141)	(0.114)	(0.046)
**Panel F. Adopted a LARC that was top-ranked method**						
IDM	0.133	0.339	0.075	0.063	–	0.154
SDM	0.038	0.386	0.138	0.286	–	0.162
SDM-IDM	−0.095	0.047	0.063	0.223	–	0.008
	(0.054)	(0.098)	(0.065)	(0.135)	–	(0.037)
**Panel G. Adopted a LARC that was second-ranked method**						
IDM	0.150	0.018	–	–	–	0.050
SDM	0.327	0.068	–	–	–	0.105
SDM-IDM	0.177	0.050	–	–	–	0.055
	(0.079)	(0.040)	–	–	–	(0.027)
**Panel H. Adopted a LARC that was third- or lower-ranked method**						
IDM	–	–	0.125	0.000	0.138	0.045
SDM	–	–	0.121	0.071	0.304	0.079
SDM-IDM	–	–	−0.004	0.071	0.166	0.034
	–	–	(0.068)	(0.067)	(0.114)	(0.024)
Obs.	112	100	98	30	52	392

SDM clients are always more likely to have discussed more methods than IDM in any subgroup (panel A), but the effects are particularly large when the IUD is ranked first (columns 1 and 3). The IUD is less popular in the study population than the implant (less than a third of IDM clients opted to discuss the IUD compared to 50% for the implant; fig. S7). SDM largely eliminated this gap. In contrast, unaware of the tailored rankings, IDM clients do not display any heterogeneity in the number of methods discussed across the distribution of top- and second-ranked methods. Panel B shows that 84% of SDM clients discussed the top-ranked method in detail, while this share is almost halved among IDM clients. SDM clients are also 8.0 pp more likely to have discussed the second-ranked method (*P* value = 0.101), especially when the implant follows the IUD in the rankings (statistically significant only at the 10% level), again pointing to the relative popularity of the implant among the study population (panel C). Clients in each counseling regime are equally likely to have discussed a method ranked third or lower (panel D). Shifting our attention to LARC adoptions, in which SDM caused a 9.7 pp increase (averaged over all prices), this effect size is similar across method rankings (panel E). Furthermore, SDM had no effect on the adoption of the top-ranked method (panel F), but it doubled the probability that a client would adopt the method ranked second for them from 5.5% in IDM to 11% in SDM (panel G). When the top two ranked methods for the client were the IUD and the implant, respectively, the share of clients adopting the implant more than doubled from 15.0% in IDM to 32.7% in SDM (*P* value < 0.01). Along with the 3.4 pp increase in the likelihood of adopting a method ranked third or lower, more than 90% of the increased LARC uptake caused by SDM comes from the adoption of IUDs and implants that were ranked second or lower for the client.

That the clients in the two counseling regimes are equally likely to have adopted the method ranked at the top for them is a positive sign for the overall quality of counseling at HGOPY with the help of the tablet-based app: It implies that the modal IDM client, who is unaware of the personalized ranking of methods, asks the right questions about the one method she discusses in detail. It further implies that the nurse counselor, who is also unaware of the app’s ranking, is able to answer these questions and carry out the discussion in such a manner that IDM clients adopt the method deemed most suitable for them as often as SDM clients. On the other hand, the substantially increased adoption of second- or lower-ranked LARCs among SDM clients is consistent with their longer search (in terms of the number of methods discussed in detail) for a method that matches their preferences: Because the expected gains from considering another method are higher with a ranked list of methods than an unranked one (*12*), SDM clients discuss more methods in detail and adopt a large share of them. Overall, the large and positive effect of SDM on LARC adoptions, especially when LARCs are offered at full price, seems to be mediated more by an increase in the number of methods discussed in detail, which is consistent with the predictions of the consumer search model of Athey and Ellison (*12*), than, say, an increase in the salience of the top-ranked method.

The relative price insensitivity of SDM clients observed in Fig. 2 is also consistent with the finding that they evaluated more methods. If clients are uncertain about the returns from adopting a LARC before counseling, i.e., they do not know how well a certain method matches their needs and preferences, then an increase in the number of methods discussed in detail should lead to reduced uncertainty. If this reduction in uncertainty is strong enough, originally marginal SDM clients (clients who would be close to indifferent between adopting versus not adopting a LARC under given prices) are no longer marginal postcounseling. As such, they become less price sensitive. In Supplemental Text section C, we present a stylized model of utility maximization with uncertainty about individual returns from adopting a LARC, which can rationalize the findings in Fig. 2.

## DISCUSSION

We conducted a randomized controlled trial of a personalized decision support app addressing informational constraints and choice architecture in the context of health technology adoption and cross-randomized it with discounts. Given a brief and neutral summary of all methods and asked to choose which method(s) they would like to discuss, IDM clients overwhelmingly discussed only one method. In contrast, the provision of personalized rankings to SDM clients and a suggestion that they should start the discussion with the top-ranked method but also that there were other promising alternatives caused a large increase in the number of methods discussed in detail, which then led to the increased adoption of LARCs from further down the ranked list. This simple tweak in counseling caused increases in LARC uptake that were as large as the effect of providing substantial discounts under IDM. As such, we not only show that LARC uptake can be substantially increased within an existing comprehensive framework for high-quality counseling (*9*) but also that such a shift might be cost-effective in preventing unintended pregnancies without causing trade-offs in client satisfaction or autonomy.

Offering discounts was effective in increasing LARC adoptions in IDM. Overall, clients aged 20 or older were almost three times more likely to adopt a LARC at full price than adolescents, but discounts eliminated this gap (table S9). Single women were more price sensitive than married (or partnered) ones. Women in the maternity or gynecology wards, most of whom had recently given birth and who were half as likely to adopt LARCs at full price, also responded favorably to discounts. These results indicate that discounts would be better targeted to vulnerable women, who are both more likely to be liquidity- or credit-constrained and for whom unintended pregnancies are costlier. Our findings are consistent with recent evidence on the effect of subsidies on contraceptive take-up [e.g., (*32*, *40*)]. They are also consistent with the use of incentives to increase the take-up of other individual preventive health measures, such as immunizations in India (*41*) or male circumcisions in South Africa (*42*).

Without a robust measure of contraceptive concordance, we cannot definitively conclude whether clients made a better choice for themselves (inclusive of adopting no method) under SDM relative to IDM. Rather, our focus is to analyze modern contraceptive uptake in response to the personalized digital counseling intervention while also providing supporting evidence related to contraceptive concordance—including method continuation, quality of care, and client satisfaction with FP services at HGOPY. The high quality of care and client satisfaction observed in SDM, combined with low rates of method discontinuations and switching, provide suggestive evidence that adoption decisions in this counseling regime are concordant with client preferences. This tentative conclusion is not unexpected because the app’s algorithm ranks methods according to many of those same preferences and SDM causes a detailed discussion of more methods.

Our study has some limitations. As discussed in the “Data” section, supporting evidence for quality of care and client satisfaction comes from participants in the adaptive trial that started immediately following the end of our study. Since this sample is exposed to exactly the same interventions and looks very similar to our study sample (table S1), we argue that it is an adequate source of data to provide supporting evidence on quality of care, client satisfaction, and method discontinuation. Similarly, we do not observe 12-month pregnancy rates (which would be reasonably defined as unintended per the inclusion criteria in our analysis of clients wishing to wait more than 12 months before becoming pregnant). However, the very high typical use effectiveness of LARCs, combined with low discontinuation rates, provide a good proxy for expected 12-month pregnancy rates. Last, our sample is composed of clients at a women and children’s hospital in Yaoundé, who differ from a random sample of women of childbearing age in the 2018 Cameroon DHS. As such, caution should be taken when applying our findings in other settings. Issues such as provider training and implementation fidelity might have particular relevance in the context of efforts to scale up.

Our study speaks to the importance of one-on-one FP service integration into all primary health care services: Clients at HGOPY, a large women and children’s hospital in Cameroon, were willing to receive counseling when approached and were responsive to the targeted interventions in our study. While increasing demand for modern contraceptives by reaching women outside of health care settings is surely important, counseling current clients of clinics represents an important opportunity for reducing unintended pregnancies and increasing birth spacing. Checklists for antenatal visits, childhood vaccinations, and postpartum and post-abortion clients could all include an item to offer FP counseling to women of childbearing age. At the same time, campaigns to reach adolescent girls and young women might be successful if they can combine high-quality counseling with affordable prices. This is consistent with recent evidence from Cameroon, which showed that providing sexual and reproductive health information to adolescents at school can decrease the incidence of teenage pregnancies (*43*).

The app that was developed as part of this project is easy to use, accepted by providers and clients alike, and can be easily integrated into existing practice as a job-support tool for counselors. As it was cheaply built on a survey platform, it would be affordable to adopt; as it has open source code, it is easy to adapt the underlying algorithm to accommodate different counseling approaches, other criteria to rank methods, and to collect different types of data on FP counseling; and as it can accommodate multiple languages with the use of drop-down menus, it would be convenient to deploy in settings with a diverse client population. It could also be adapted for use as a decision-support tool for clients, either in the privacy of their home (in the form of a phone app or an online tool) or on a tablet in the waiting rooms of clinics. We can foresee two issues in the effective deployment of the app in real-life clinical settings: First, as a job-support tool, it would be ideally used by well-trained FP providers, but the marginal training required to use the app is minimal.

Second, the app is not designed to be part of a medical information system: If a clinic or a health system wanted to use it to link client data across FP visits over time and merge it with other relevant data for the client, then it would need to be professionally adapted for those purposes. Last, our findings complement several broader trends. Digital tools are becoming ubiquitous, including for health technology, and it is clear that even in our setting—personal discussions around a sensitive topic—they can be well accepted by both clients and providers alike. We see notable impacts from personalized recommendations, which are in part made feasible exactly due to digitization. The potential social welfare benefits of individual rankings may extend to a wide variety of environments, especially online, going beyond health to education and household finance.

## Supplementary Materials

### This PDF file includes:

Figs. S1 to S9 Tables S1 to S10 Supplemental Text, sections C and D

## References

[1] E. Sully, A. Biddlecom, J. E. Darroch, T. Riley, L. S. Ashford, N. Lince-Deroche, L. Firestein, R. Murro, Adding it up: Investing in sexual and reproductive health 2019, *Tech. Rep.*, Guttmacher Institute, Washington DC (2020).

[2] S. Baird, C. McIntosh, B. Özler, Cash or condition? Evidence from a cash transfer experiment. Q. J. Econ. 126, 1709–1753 (2011).

[3] E. A. DeFranco, L. M. Seske, J. M. Greenberg, L. J. Muglia, Influence of interpregnancy interval on neonatal morbidity. Am. J. Obstet. Gynecol. 212, 386.e1–386.e9 (2015). 10.1016/j.ajog.2014.11.017 25460837

[4] J. J. Frost, A. Sonfield, M. R. Zolna, L. B. Finer, Return on investment: A Fuller assessment of the benefits and cost savings of the us publicly funded family planning program. Milbank Q. 92, 696–749 (2014). 25314928 10.1111/1468-0009.12080 PMC4266172

[5] D. E. Bloom, D. Canning, G. Fink, J. E. Finlay, Fertility, female labor force participation, and the demographic dividend. J. Econ. Growth 14, 79–101 (2009).

[6] D. E. Bloom, M. Kuhn, K. Prettner, The contribution of female health to economic development. Econ. J. 130, 1650–1677 (2020).

[7] World Health Organization, Trends in maternal mortality 2000 to 2017: Estimates by WHO, UNICEF, UNFPA, World Bank Group and the United Nations Population Division, *Tech. Rep.*, World Health Organization (2019).

[8] D. Maggio, M. Karra, D. Canning, Family planning and children’s human capital: Experimental evidence from urban Malawi. Tech. Rep., 1–48 (2022). 10.1215/00703370-11581796 39324834

[9] K. Holt, C. Dehlendorf, A. Langer, Defining quality in contraceptive counseling to improve measurement of individuals' experiences and enable service delivery improvement. Contraception 96, 133–137 (2017). 28645786 10.1016/j.contraception.2017.06.005

[10] L. Senderowicz, Contraceptive autonomy: Conceptions and measurement of a novel family planning indicator. Stud. Fam. Plann. 51, 161–176 (2020). 32358789 10.1111/sifp.12114

[11] K. Newman, C. Feldman-Jacobs, Family Planning and Human Rights: What's the Connection and Why is It so Important? *Tech. Rep.,* Population Reference Bureau (2015).

[12] S. Athey, G. Ellison, Position auctions with consumer search. Q. J. Econ. 126, 1213–1270 (2011).

[13] A. Ghose, P. G. Ipeirotis, B. Li, Examining the impact of ranking on consumer behavior and search engine revenue. Manage. Sci. 60, 1632–1654 (2014).

[14] R. Epstein, R. E. Robertson, The search engine manipulation effect (SEME) and its possible impact on the outcomes of elections. Proc. Natl. Acad. Sci. U.S.A. 112, E4512–E4521 (2015). 26243876 10.1073/pnas.1419828112 PMC4547273

[15] A. Allam, P. J. Schulz, K. Nakamoto, The impact of search engine selection and sorting criteria on vaccination beliefs and attitudes: Two experiments manipulating google output. J. Med. Internet Res. 16, e100 (2014). 24694866 10.2196/jmir.2642 PMC4004139

[16] M. Karra, K. Zhang, User-centered counseling and male involvement in contraceptive decision making: Protocol for a randomized controlled trial. JMIR Res. Protoc. 10, e24884 (2021). 33818398 10.2196/24884 PMC8056297

[17] E. J. Johnson, M. Steffel, D. G. Goldstein, Making better decisions: From measuring to constructing preferences. Health Psychol. 24, S17–S22 (2005). 16045413 10.1037/0278-6133.24.4.S17

[18] J. Carpenter, E. Huet-Vaughn, P. H. Matthews, A. Robbett, D. Beckett, J. Jamison, Choice architecture to improve financial decision making. Rev. Econ. Stat. 103, 102–118 (2021).

[19] I. Bohnet, A. Van Geen, M. Bazerman, When performance trumps gender bias: Joint vs. separate evaluation. Manage. Sci. 62, 1225–1234 (2016).

[20] S. Athey, J. C. Castillo, B. Chandar, Service quality in the gig economy: Empirical evidence about driving quality at uber. SSRN Electron. J., 1–51 (2019).

[21] T. N. Hubbard, Information, decisions, and productivity: On-board computers and capacity utilization in trucking. Am. Econ. Rev. 93, 1328–1353 (2003).

[22] A. Awaysheh, J. Wilcke, F. Elvinger, L. Rees, W. Fan, K. L. Zimmerman, Review of medical decision support and machine-learning methods. Vet. Pathol. 56, 512–525 (2019). 30866728 10.1177/0300985819829524

[23] O. Obasola, I. Mabawonku, I. Lagunju, A review of e-health interventions for maternal and child health in sub-sahara Africa. Matern. Child Health J. 19, 1813–1824 (2015). 25652059 10.1007/s10995-015-1695-0

[24] M. L. Gilliam, S. L. Martins, E. Bartlett, S. Q. Mistretta, J. L. Holl, Development and testing of an iOS waiting room app for contraceptive counseling in a title X family planning clinic. Am. J. Obstet. Gynecol. 211,481.481.e1–481.e8 (2014). 10.1016/j.ajog.2014.05.034 24881829

[25] K. Church, C. E. Warren, I. Birdthistle, G. B. Ploubidis, K. Tomlin, W. Zhou, J. Kimani, T. Abuya, C. Ndwiga, S. Sweeney, S. H. Mayhew, Impact of integrated services on HIV testing: A nonrandomized trial among kenyan family planning clients. Stud. Fam. Plann. 48, 201–218 (2017). 28470971 10.1111/sifp.12022 PMC5518195

[26] E. K. Wilson, K. E. Krieger, H. P. Koo, A. M. Minnis, K. Treiman, Feasibility and acceptability of a computer-based tool to improve contraceptive counseling. Contraception 90, 72–78 (2014). 24815097 10.1016/j.contraception.2014.02.027

[27] C. Dehlendorf, J. Fitzpatrick, J. Steinauer, L. Swiader, K. Grumbach, C. Hall, M. Kuppermann, Development and field testing of a decision support tool to facilitate shared decision making in contraceptive counseling. Patient Educ. Couns. 100, 1374–1381 (2017). 28237522 10.1016/j.pec.2017.02.009 PMC5985808

[28] C. Dehlendorf, J. Fitzpatrick, E. Fox, K. Holt, E. Vittinghoff, R. Reed, M. P. Campora, A. Sokoloff, M. Kuppermann, Cluster randomized trial of a patient-centered contraceptive decision support tool, my birth control. Am. J. Obstet. Gynecol. 220, 565.e1–565.e12 (2019). 10.1016/j.ajog.2019.02.015 30763545

[29] N. Ashraf, E. Field, J. Lee, Household bargaining and excess fertility: An experimental study in Zambia. Am. Econ. Rev. 104, 2210–2237 (2014).

[30] B. Bellows, C. Bulaya, S. Inambwae, C. L. Lissner, M. Ali, A. Bajracharya, Family planning vouchers in low and middle income countries: A systematic review. Stud. Fam. Plann. 47, 357–370 (2016). 27859338 10.1111/sifp.12006 PMC5434952

[31] S. Anukriti, C. Herrera-Almanza, M. Karra, Women’s access to family planning: Experimental evidence on the role of peers and vouchers. Tech. rep., (2021).

[32] M. Bailey, V. W. Lang, I. Vrioni, L. Bart, D. Eisenberg, P. Fomby, J. Barber, V. K. Dalton, How subsidies affect contraceptive use among low-income women in the U.S.: A randomized control trial, Tech. rep., University of California, Los Angeles (2021).

[33] T. Rau, M. Sarzosa, S. Urzúa, The children of the missed pill. J. Health Econ. 79, 102496 (2021). 34399313 10.1016/j.jhealeco.2021.102496 PMC8496187

[34] R. Mestad, G. Secura, J. E. Allsworth, T. Madden, Q. Zhao, J. F. Peipert, Acceptance of long-acting reversible contraceptive methods by adolescent participants in the contraceptive CHOICE project. Contraception 84, 493–498 (2011). 22018123 10.1016/j.contraception.2011.03.001 PMC3505875

[35] J. M. Lindo, A. Packham, How much can expanding access to long-acting reversible contraceptives reduce teen birth rates? Am. Econ. J. Econ. Policy 9, 348–376 (2017).

[36] M. Shah, J. Seager, J. Montalvao, M. Goldstein, Sex, Power, and Adolescence: Intimate Partner Violence and Sexual Behavior, *Tech. rep.,* National Bureau of Economic Research (2023).

[37] D. Hubacher, H. Spector, C. Monteith, P.-L. Chen, C. Hart, Long-acting reversible contraceptive acceptability and unintended pregnancy among women presenting for short-acting methods: A randomized patient preference trial. Am. J. Obstet. Gynecol. 216, 101–109 (2017). 27662799 10.1016/j.ajog.2016.08.033 PMC5479328

[38] J. Trussell, Contraceptive failure in the United States. Contraception 83, 397–404 (2011). 21477680 10.1016/j.contraception.2011.01.021 PMC3638209

[39] A. Jain, K. Aruldas, A. Mozumdar, E. Tobey, R. Acharya, Validation of two quality of care measures: Results from a longitudinal study of reversible contraceptive users in India. Stud. Fam. Plann. 50, 179 (2019). 31120148 10.1111/sifp.12093

[40] M. Karra, D. Maggio, M. Guo, B. Ngwira, D. Canning, The causal effect of a family planning intervention on women's contraceptive use and birth spacing. Proc. Natl. Acad. Sci. U.S.A. 119, e2200279119 (2022). 35609202 10.1073/pnas.2200279119 PMC9295775

[41] A. Banerjee, A. Chandrasekhar, S. Dalpath, E. Duflo, J. Floretta, M. Jackson, H. Kannan, F. Loza, A. Sankar, A. Schrimpf, M. Shrestha, Selecting the most effective nudge: Evidence from a large-scale experiment on immunization, *Tech. rep.,* National Bureau of Economic Research (2021).

[42] W. Friedman, N. Wilson, Can nudging overcome procrastinating on preventive health investments? Econ. Human Biol. 45, 101040 (2022). 35176570 10.1016/j.ehb.2021.101040

[43] P. Dupas, E. Huillery, J. Seban, Risk information, risk salience, and adolescent sexual behavior: Experimental evidence from Cameroon. J. Econ. Behav. Organ. 145, 151–175 (2018).

