# Supplementary material for: Can personalized digital counseling improve consumer search for modern contraceptive methods?
Source: Sci Adv. 2023 Oct 6;9(40):eadg4420. doi: 10.1126/sciadv.adg4420 (PMC10558117; doi:10.1126/sciadv.adg4420)
Supplement: Supplementary file 1 — Figs. S1 to S9 Tables S1 to S10 Supplemental Text, sections C and D [file sciadv.adg4420_sm.pdf]

Science Advances
AAAS

Supplementary Materials for

Can personalized digital counseling improve consumer search for modern contraceptive methods?

Susan Athey *et al.*

Corresponding author: Berk Özler, bozler@worldbank.org

Sci. Adv. **9**, eadg4420 (2023)
DOI: 10.1126/sciadv.adg4420

This PDF file includes:

Figs. S1 to S9
Tables S1 to S10
Supplemental Text, sections C and D

A Appendix tables

Table S1: Client characteristics.

	(1)<br>**Pilot Sample**<br>Mean/(SD)	(2)<br>**Follow-up Sample**<br>Mean/(SD)	(3)<br>**DHS: Yaoundé**<br>Mean/(SD)
Age	29.27(7.03)	29.40(6.43)	26.98(9.05)
Adolescent	0.09	0.06	0.28
BMI	27.54(4.86)	27.16(4.76)	25.09(3.76)
Unmarried couple cohabiting	0.37	0.33	0.16
Married	0.33	0.36	0.23
Education: Tertiary	0.42	0.50	0.21
Education: Secondary	0.23	0.23	0.64
Education: Primary/Lower sec.	0.33	0.26	0.13
Salaried employee	0.32	0.35	0.18
Self-employed	0.21	0.12	0.29
Student	0.21	0.16	0.31
Pregnancies, total	3.69(2.33)	3.52(2.26)	1.86(2.03)
Children alive today	2.75(1.81)	2.55(1.72)	1.75(1.86)
Ever gave birth (live or still)	0.94	0.92	0.63
Gave birth <=3 months	0.56	0.58	0.05
Wants no more children	0.25	0.23	0.28
Wait 1 to 3 yrs before next preg.	0.36	0.42	0.16
Wait >3 years before next preg.	0.39	0.35	0.15
Currently using a LARC	0.03	0.04	0.05
Currently using a SARC	0.04	0.02	0.06
Currently using other method	0.05	0.05	0.24
MiM	0.49	0.53	-
Number of observations	784	1,141	1,067

Note: This table shows client characteristics in the study sample (Pilot sample), the follow-up interview sample - which was recruited during the adaptive phase of the study, immediately after the pilot -, and the DHS 2018 Yaoundé Stratum.

Table S2: Method mix.

	(1)	(2)	(3)	(4)	(5)	(6)
	**Current method**		**Method in mind**		**Method adopted**	
	N	%	N	%	N	%
**None**	**689**	**87.88**	**342**	**43.62**	**237**	**30.23**
LARC	**26**	**3.32**	**358**	**45.66**	**342**	**43.62**
IUD	3	0.38	99	12.63	101	12.88
Implant	23	2.93	259	33.04	241	30.74
SARC	**29**	**3.70**	**62**	**7.91**	**35**	**4.46**
Pill	11	1.40	18	2.30	17	2.17
Injectable	18	2.30	44	5.61	18	2.30
Other	**40**	**5.10**	**22**	**2.81**	**170**	**21.68**
LAM	1	0.13	5	0.64	153	19.52
Male/Female condoms	36	4.59	8	1.02	12	1.53
Traditional or other	3	0.38	9	1.15	5	0.64
**Total**	**784**	**100.00**	**784**	**100.00**	**784**	**100.00**

Notes: This table shows the method mix amongst the clients who visit the hospital included in the study sample; columns 1 and 2 show the number and fraction of clients who are currently using each method at the time of their first consultation; columns 2 and 3 shows which method the 442 clients had in mind during their consultation, i.e. the method they wanted to adopt or renew without discussing other methods (see Figure 1); column 5 and 6 show the method that was ultimately adopted by the clients, noting that LAM and condoms can be used concurrently with other methods and are thus counted as the method adopted when they are used as the primary method of contraception; the IUD refers to the copper IUD, LAM refers to lactational amenorrhea method, traditional method or other encompasses all other methods and primarily consists of the calendar method and coitus interruptus method.

Table S3: Balance table over LARC prices

	(1) Control	(2) LARC price: Discounted	(3)
	Mean/(SD)	Mean/(SD)	Diff 1-2 (p-value)
Dep.: Family Planning	0.59	0.57	0.61
Age	28.79/(7.44)	29.39/(6.92)	0.34
Adolescent	0.10	0.08	0.39
BMI	27.33/(4.92)	27.59/(4.85)	0.56
Single	0.35	0.28	0.12
Unmarried couple cohabiting	0.34	0.38	0.40
Married	0.31	0.34	0.55
Education: Tertiary	0.38	0.43	0.31
Education: Secondary	0.27	0.22	0.20
Education: Primary/Lower sec.	0.35	0.32	0.59
Education: None	0.00	0.03	0.04
Salaried employee	0.27	0.33	0.16
Self-employed	0.19	0.21	0.45
Student or apprentice	0.26	0.22	0.29
Domestic activities	0.26	0.21	0.27
Pregnancies, total	3.66/(2.45)	3.70/(2.30)	0.86
Children alive today	2.63/(1.80)	2.78/(1.81)	0.35
Ever gave birth (live or still)	0.94	0.94	0.87
Wants no more children	0.22	0.26	0.35
Wait 1 to 3 yrs before next preg.	0.40	0.35	0.21
Wait >3 years before next preg.	0.37	0.39	0.70
Currently using a LARC	0.03	0.04	0.56
Currently using a SARC	0.03	0.04	0.72
Currently using other method	0.03	0.06	0.23
Method in mind	0.50	0.49	0.80
Test of joint orthogonality, F-stat			0.86
p-value			0.65
N	156	628	

Notes: the 'Difference' columns show the p-value from a t-test of the difference in means between the two indicated groups; the F-test of joint-orthogonality (F-stat/p-value) tests that all the coefficients are jointly equivalent to zero when regressing the set of variables shown in this table on a group indicator; Standard deviation in parentheses for non-binary variables.

Table S4: Balance table over the counselling style

	(1) **IDM**	(2) **SDM**	(3)
	Mean/(SD)	Mean/(SD)	Diff. (p-value)
Dep.: Family Planning	0.50	0.48	0.65
Age	28.67/(7.06)	27.84/(7.18)	0.24
Adolescent	0.11	0.12	0.69
BMI	27.49/(4.69)	27.45/(4.71)	0.93
Single	0.33	0.39	0.18
Unmarried couple cohabiting	0.40	0.37	0.53
Married	0.28	0.24	0.45
Education: Tertiary	0.39	0.37	0.75
Education: Secondary	0.28	0.21	0.14
Education: Primary/Lower sec.	0.32	0.39	0.12
Education: None	0.02	0.03	0.71
Salaried employee	0.28	0.25	0.60
Self-employed	0.20	0.22	0.62
Student or apprentice	0.28	0.27	0.86
Domestic activities	0.21	0.24	0.54
Pregnancies, total	3.42/(2.38)	3.35/(2.28)	0.76
Children alive today	2.61/(1.84)	2.39/(1.77)	0.23
Ever gave birth (live or still)	0.94	0.92	0.52
Wants no more children	0.19	0.23	0.39
Wait 1 to 3 yrs before next preg.	0.45	0.40	0.32
Wait >3 years before next preg.	0.36	0.37	0.77
Currently using a LARC	0.00	0.01	0.15
Currently using a SARC	0.03	0.03	0.96
Currently using other method	0.02	0.04	0.22
Test of joint orthogonality, F-stat			0.93
p-value			0.56
N	202	197	

Notes: the 'Difference' columns show the p-value from a t-test of the difference in means between the two indicated groups; the F-test of joint-orthogonality (F-stat/p-value) tests that all the coefficients are jointly equivalent to zero when regressing the set of variables shown in this table on a group indicator; Standard deviation in parentheses for non-binary variables.

Table S5: Prices in nearby Health Centers

	(1) HGOPY	(2) Hôpital Général	(3) Centre Medical Saint Luc De Ngousso	(4) Centre de Santé Ste Glanie	(5) Centre de Santé Joss	(6) Centre de Santé Humani-taire	(7) Centre de Santé Grace Divine Ngousso
IUD	4000	2500	5000	5000	3000	15000	5000
Implant	4000	2500	5000	7000	5000	7000	5000
Injectable	1250	1000	1500	1500	1500	2000	1500
Pills	1500	500	1000	1000	1000	1000	1000
Removals	0	5000	5000	5000	5000	5000	5000
Consultation	0	500-1000	500-1000	500-1000	500-1000	500-1000	500-1000
Distance to HGOPY (meters)	-	399	3379	2571	1146	982	1605
Walking time (minutes)	-	4.40	27.73	34.25	15.08	12.78	19.70
Driving time (minutes)	-	1.30	8.50	6.90	3.20	2.47	4.58

Notes: This table presents prices of LARCs, SARCs, Removals and Consultation cost in health centers that are close to the Yaoundé Gynaecology, Obstetrics and Pediatrics Hospital (HGOPY); The data was personally collected by a nurse consultant with Family Planning experience; column (1) uses the highest price that was offered to clients during the study at HGOPY; Distance to HGOPY is in meters and is calculated using Gooogle's Distance Matrix API; Walking and driving time are measured using predictive traffic information.

Table S6: Rankings produced by the app's internal algorithm

	(1) Pos. 1	(2) Pos. 2	(3) Pos. 3	(4) Pos. 4	(5) Pos. 5
IUD	0.54	0.31	0.08	0.07	0.00
Implant	0.33	0.30	0.24	0.13	0.00
Pill	0.05	0.24	0.38	0.25	0.18
Injectable	0.00	0.00	0.18	0.44	0.78
LAM	0.08	0.16	0.12	0.11	0.04
Total	1.00	1.00	1.00	1.00	1.00

Notes: This table presents the positions produced by the app's internal algorithm; Each column represents the percent of occurrence of each contraceptive within each position.

Table S7: Impacts Over Time – Method at 16 week follow-up survey

Adopted method during counselling	Method at 16 week follow-up survey							
	Neither		LARC		SARC		Total	
	N	%	N	%	N	%	N	%
Neither	302	77.2	71	18.2	18	4.6	391	100.0
LARC	9	7.6	109	92.4	0	0.0	118	100.0
SARC	7	50.0	0	0.0	7	50.0	14	100.0
Total	318	60.8	180	34.4	25	4.8	523	100.0

Notes: The data shown in this table covers a sample of clients enrolled in the adaptive experiment following this study between the 19th of January 2021 and the 29th of June 2022.

Table S8: Quality of care and client satisfaction by counselling style.

	(1) **MiM**	(2) **IDM**	(3) **SDM**	(4) Diff (2)-(3))
Variable	Mean	Mean	Mean	(p-value)
Panel A: Quality of care measures as per Jain et al. (2019)				
**Quality of care index**	**67.05**	**67.44**	**68.87**	**0.48**
**Method selection sub-index**	**91.54**	**90.05**	**93.27**	**0.18**
Asked about desire for another child	95.28	92.68	96.28	
Asked about preferred timing for another child	87.73	85.93	89.90	
Asked about previous FP experience	89.78	86.90	90.94	
Asked about preferred FP method	93.39	94.70	95.97	
Given information about different FP methods	92.21	98.49	98.56	
**Effective use sub-index**	**88.97**	**85.29**	**89.34**	**0.26**
Told about side effects or problems with selected method	88.33	94.91	89.60	
Told how to manage side effects or problems with selected method	88.28	80.93	88.22	
Told about warning signs for the selected method	90.29	80.03	90.20	
**Continuity of care sub-index**	**77.46**	**78.17**	**85.80**	**0.12**
Told about possibility of switching to another method	77.46	78.17	85.80	
**Audio and Visual Privacy sub-index**	**10.23**	**16.23**	**7.07**	**0.02**
Consulted where nobody could see them	10.56	16.21	7.05	
Consulted where nobody could hear them	9.89	16.26	7.09	
Panel B: Client satisfaction with services				
Satisfied with FP services in general	88.96	90.00	89.40	0.86
Satisfied with FP consultation	90.69	93.36	94.29	0.73
Likely to return for FP services	92.18	94.35	93.98	0.87
N	641	175	368	

Notes: The data shown in this table uses data from the 2-week follow-up interviews for the follow-up cohort - i.e. the sample of clients counselled between the 19th of January 2021 and the 29th of June 2022. These clients were enrolled into the study during the adaptive experiment phase, so all estimates are weighted by the inverse probability of assignment of their assigned treatment arm. The 'Diff' column, column 4, shows the p-value from a t-test of the difference in means between the IDM and SDM groups. The quality of care index is calculated following Jain et al. (2019), where each sub-index is obtained by averaging over the components within the domain and the overall index is obtained by averaging across the four domain domain indices, equally weighted; the audio and visual privacy index reflects the respectful care domain from Jain et al. The measures of client satisfaction with the services provided indicate the fraction of clients who are satisfied, or very satisfied with FP services and general and with the FP consultation specifically, as well as the clients who are likely, or very likely to return to the study hospital for FP services in the future.

Table S9: Heterogeneity of impacts of price discounts.

	(1)	(2)	(3)	(4)	(5)	(6)
	**Department**		**Age group**		**Marital status**	
	FP	Mat./Gyn.	Age≥20	Age<20	Mar./Coh.	Single
LARC price: Discounted	0.162	0.128	0.120	0.346	0.113	0.199
	(0.058)	(0.058)	(0.045)	(0.109)	(0.053)	(0.070)
Control mean w/n group	0.402	0.203	0.343	0.125	0.353	0.259
Discount=Full w/n group (p-val.)	0.005	0.028	0.008	0.002	0.034	0.005
Discount=Discount b/w groups (p-val.)	0.672		0.055		0.328	
Obs. in group	448	336	717	67	551	233
Obs.	784		784		784	

Notes: Robust standard error in parentheses; Discounted prices for LARCs were offered at CFA 2,000, 1,000, 150, and free; Discounted prices for SARCs were offered at no cost; all sets of columns show the estimated coefficients for the group-specific impacts of LARC prices estimated from a single regression on the outcome on LARC prices interacted with a group indicator; regressions shown in columns 1 and 2 report results from interacting LARC prices with the department where the client was received as the group indicator, separated as Family Planning and Maternity/Gynecology+others; regressions in columns 3 and 4 use an indicator for the age group; regression in columns 5 and 6 use an indicator for marital status, separated as Married/Cohabiting and Single.

B Appendix figures

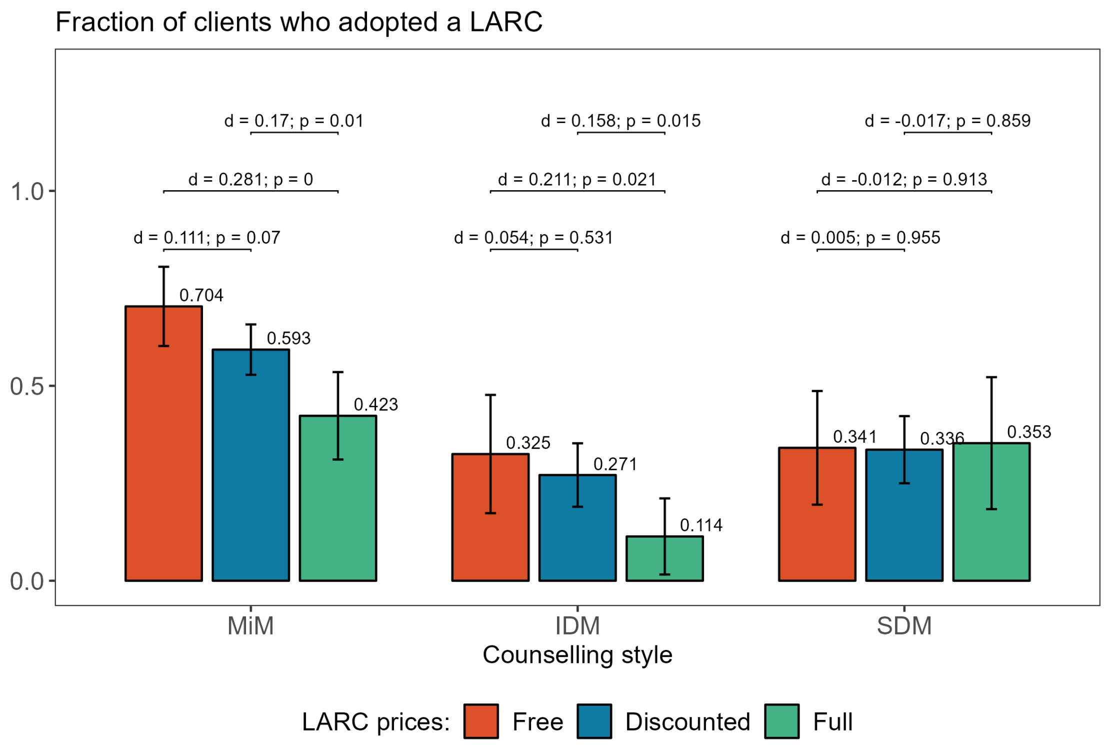

Figure S1: Impact of LARC prices on LARC adoptions: The figure shows the fraction of clients who adopted a LARC during the study period under *Free*, *Discounted*, and *Full* priced LARCs across counseling styles. The lines above the bars show the estimate (d) and p-value (p) from a t-test of the difference in means between the two indicated groups.

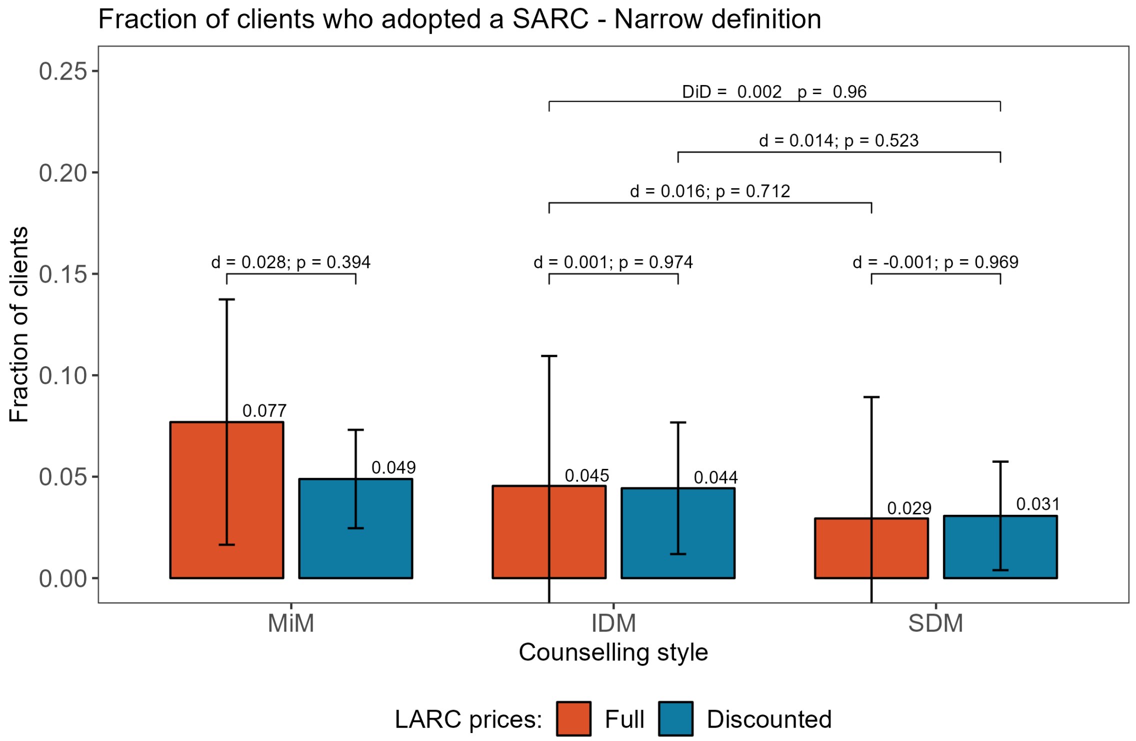

Figure S2: Impact of LARC prices on SARC adoptions: The figure shows the fraction of clients who adopted a SARC under *Discounted*, and *Full* priced LARCs across counseling styles. The "narrow" definitions refers to SARCs as defined in our study and includes the Pill (POP or COC) and the Injectable. The lines above the bars show the estimate (d) and p-value (p) from a t-test of the difference in means between the two indicated groups. The bar labeled *DiD* indicates the difference-in-differences estimate between the two randomized counseling interventions (*IDM-SDM*) and LARC price discounts (*Full-Discounted*).

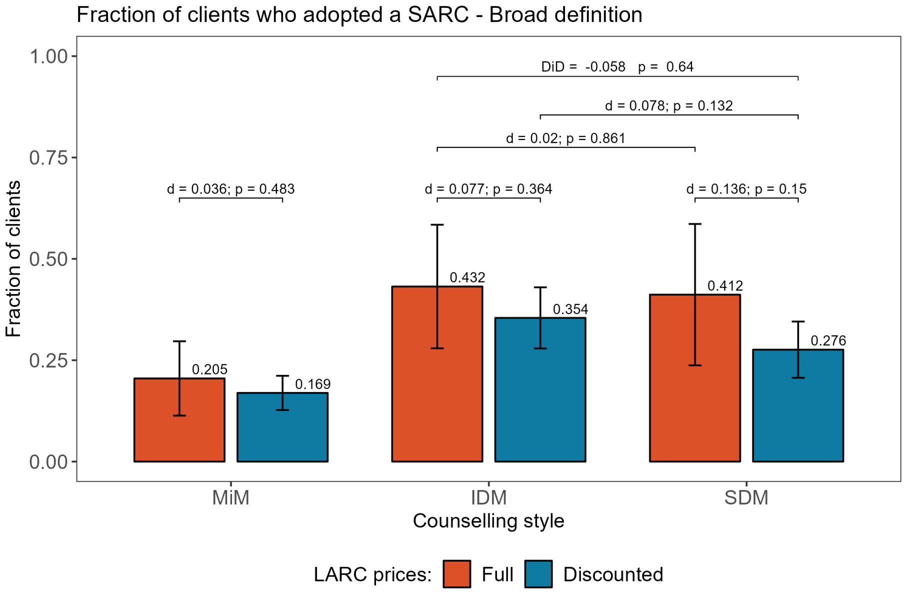

Figure S3: Impact of LARC prices on SARC adoptions: The figure shows the fraction of clients who adopted a SARC under *Discounted*, and *Full* priced LARCs across counseling styles. The "broad" definitions refers to a definition of SARCs which includes the Pill (POP or COC), the Injectable, Condoms, the Standard Days Methods, and Emergency contraception. The lines above the bars show the estimate (d) and p-value (p) from a t-test of the difference in means between the two indicated groups. The bar labeled *DiD* indicates the difference-in-differences estimate between the two randomized counseling interventions (*IDM-SDM*) and LARC price discounts (*Full-Discounted*).

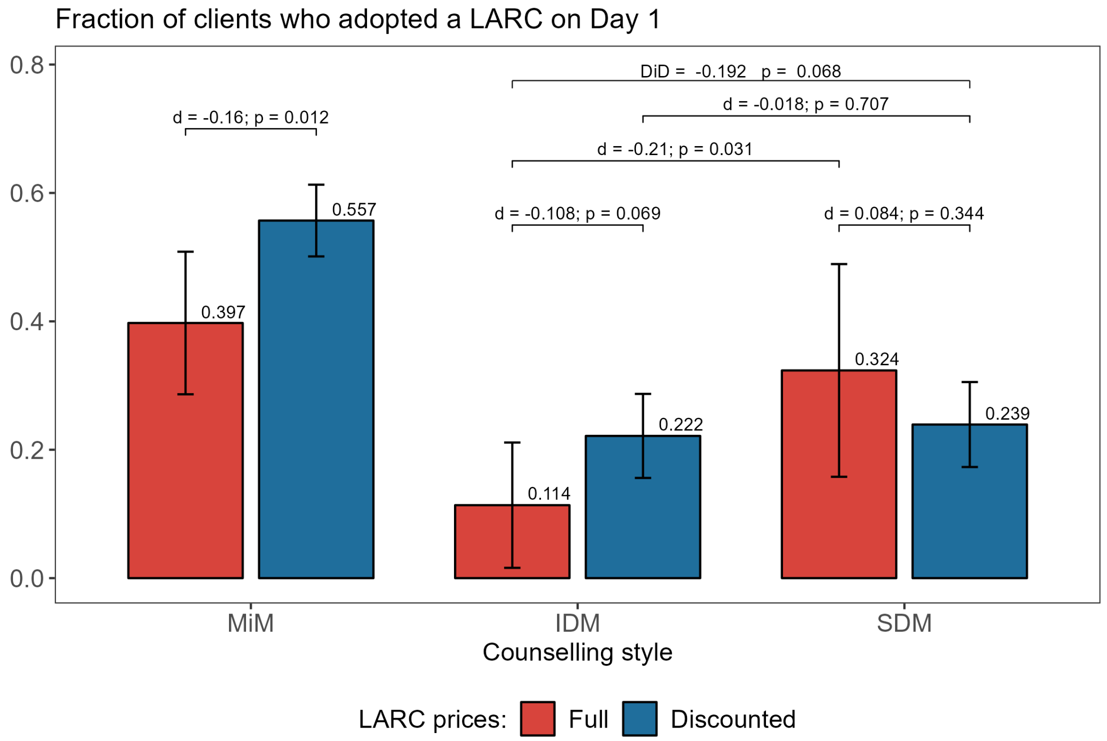

Figure S4: Impact of LARC prices on LARC adoptions, after the first visit: The figure shows the fraction of clients who adopted a LARC on the day of their first counselling session during the study period, *Discounted*, and *Full* priced LARCs across counseling styles. The lines above the bars show the estimate (d) and p-value (p) from a t-test of the difference in means between the two indicated groups. The bar labeled *DiD* indicates the difference-in-differences estimate between the two randomized counseling interventions (*IDM-SDM*) and LARC price discounts (*Full-Discounted*).

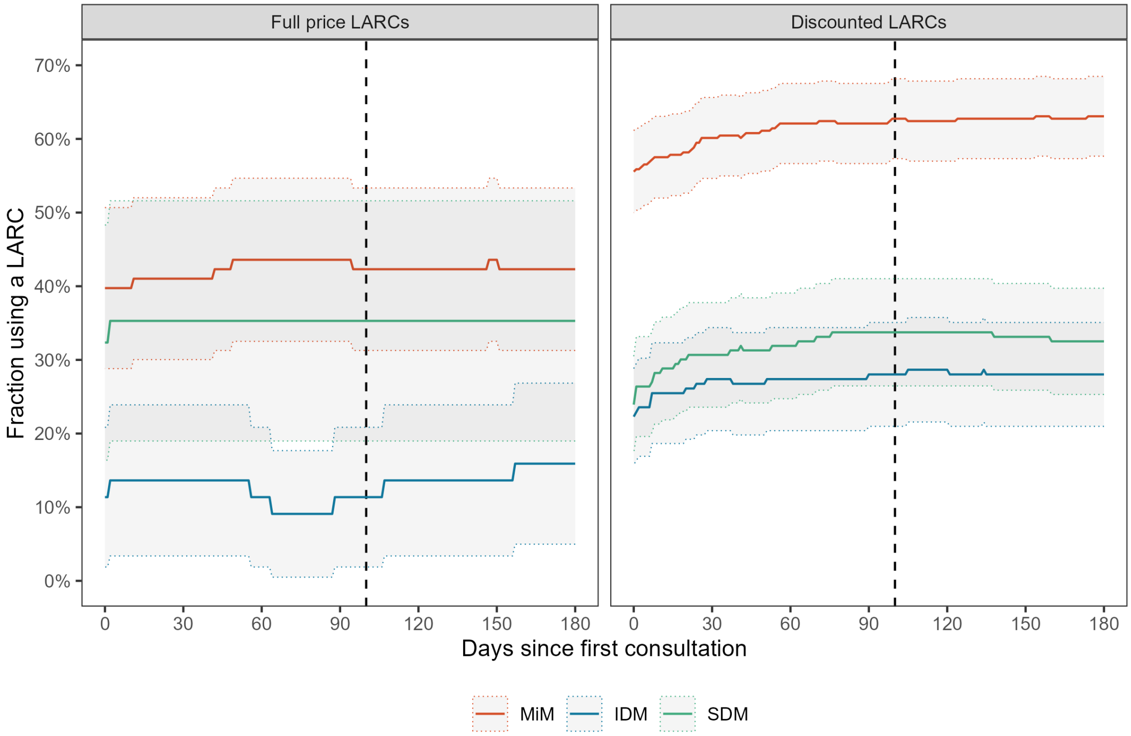

Figure S5: Impact on LARC adoptions over time: The figure shows the share of clients who were using a LARC at any point in time between their first counselling session during the study period and the following 180 days. The data is based on administrative data collected at the hospital, tracking each client across visits.

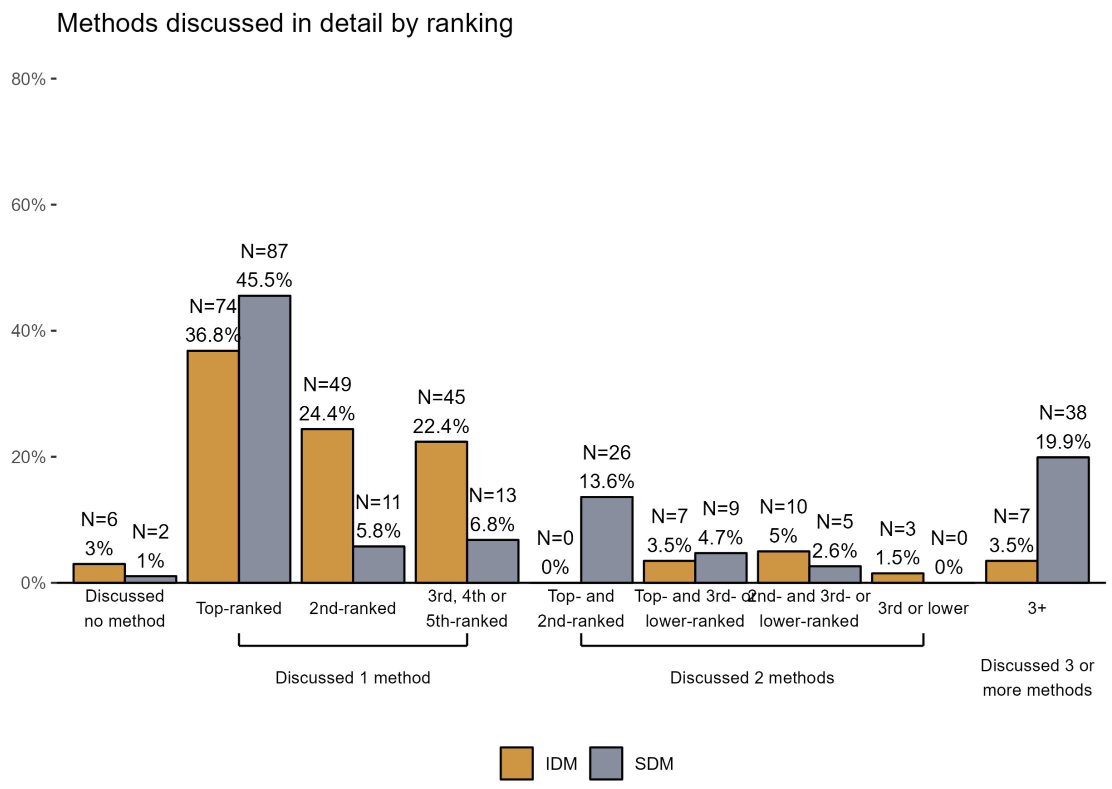

Figure S6: Distribution of the number of methods discussed combined with rankings by SDM and IDM regimes: The figure illustrates the distribution of the number of methods discussed and their rankings for clients under both regimes. Discussion is divided into four categories: discussed no methods, discussed one method, discussed two methods, and discussed three or more methods.

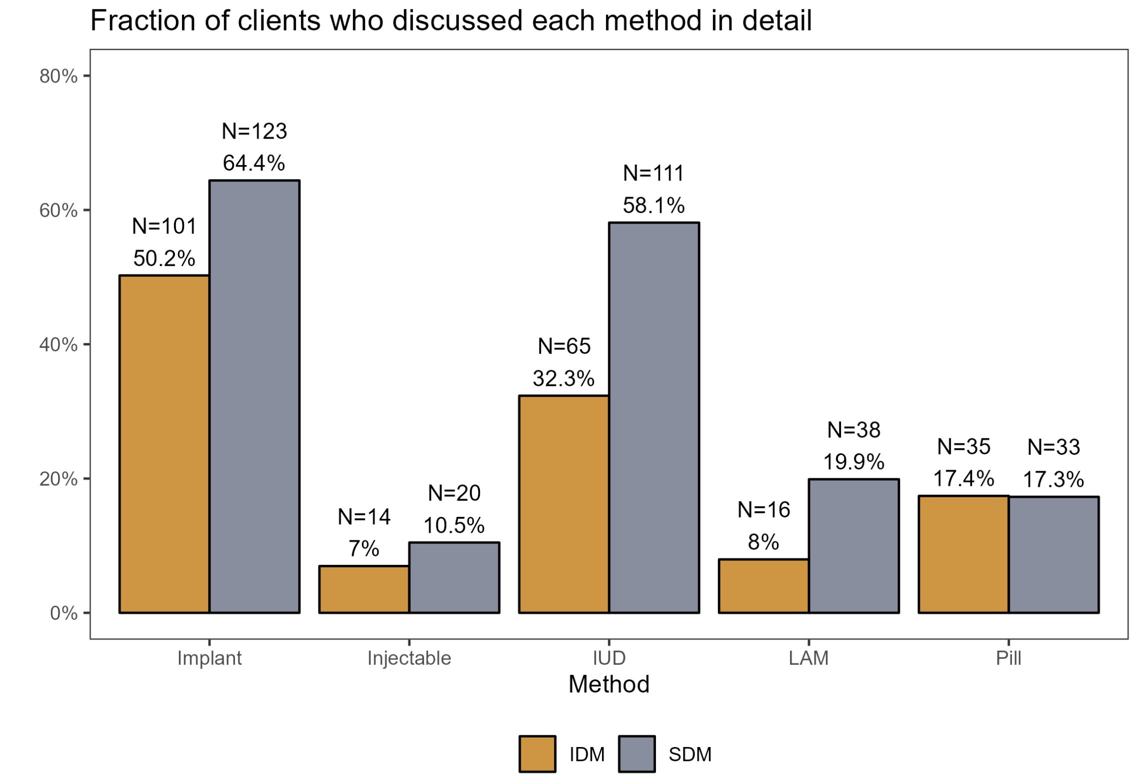

Figure S7: Distribution of method discussion by SDM and IDM regimes: The figure provides a breakdown of the fraction of clients who discussed each method in detail under both regimes.

C A Simple Model of Contraceptive Method Adoption and Price Effects for LARCs: *SDM* vs. *IDM*

C.1 Individual Problem

Consider a world where individuals have utility over consumption and whether or not they adopt a LARC: $u(c) + ql$ where c denotes consumption, $l \in \{0, 1\}$ denotes whether or not the individual adopts the LARC (for simplicity we assume that there is just one type of LARC), and q denotes the return to the LARC. Moreover, assume that if the individual does not adopt the LARC, she adopts a non-modern method which is normalized to give utility of 0 (i.e., everyone's best alternative to the LARC is a non-modern method; this assumption can easily be relaxed but seems applicable in our setting given the low uptake of SARCs). Next, assume that there are two types of individuals: individuals with a high return to the LARC, $q = q_h$, and individuals with a low return to the LARC, $q = q_l < q_h$. However, individuals do not know their type. Instead they assume they are type q_h with probability $\theta \in [0, 1]$. Furthermore, assume income is endowed and denoted by y, so that for a given price of the LARC, p, consumption is given by $c = y - pl$.[1] Finally, assume for simplicity that individuals have common income y (this, too, can easily be relaxed) but do not have common beliefs over their type; in other words, individuals are indexed by their (perceived) probability that they have a high return to the LARC, θ. An individual with probability θ facing price p solves:

$$\max_{l \in \{0,1\}} l \left[\theta \left(u(y-p) + q_h\right) + (1-\theta)\left(u(y-p) + q_l\right)\right] + (1-l)u(y)$$

Let $\tilde{\theta}(p)$ denote the individual who is indifferent between adopting the LARC vs. not under price p. Implicitly, $\tilde{\theta}(p)$ is defined as follows:

$$\tilde{\theta}\left(u(y-p) + q_h\right) + (1-\tilde{\theta})\left(u(y-p) + q_l\right) = u(y) \tag{1}$$

By the Implicit Function Theorem, $\partial\tilde{\theta}(p)/\partial p = u_c(y-p)/(q_h - q_l)$; thus $\partial\tilde{\theta}(p)/\partial p > 0$ by the standard assumption that utility is strictly increasing in consumption, $u_c > 0$. Notably, because the LHS of Equation 1 is strictly increasing in θ, an individual will adopt the LARC under price p iff $\theta \geq \tilde{\theta}(p)$. Thus, for a given (cumulative) distribution of types, $F(\theta)$, the share adopting the LARC under price p, $S(p)$, is given by:

$$S(p) = \int_{\tilde{\theta}(p)}^{1} dF(\theta) = 1 - F\left(\tilde{\theta}(p)\right) \tag{2}$$

Note, if the price is low enough s.t. $\tilde{\theta}(p) \leq 0$, then $S(p) = 1$, i.e., everyone adopts the LARC. Conversely if the price is high enough s.t. $\tilde{\theta}(p) > 1$, then $S(p) = 0$, i.e., no one adopts the LARC.

Next, consider what happens to the share adopting the LARC as we raise the price:[2]

[1] For simplicity, we assume the non-modern method is free; this can easily be relaxed.

[2] For simplicity, we assume $F(\theta)$ admits a density function $f(\theta)$.

$$\frac{\partial S(p)}{\partial p} = -\frac{\partial \tilde{\theta}(p)}{\partial p} f(\tilde{\theta}(p)) \leq 0$$

The inequality comes from the fact that $\partial\tilde{\theta}(p)/\partial p > 0$. Thus, as to be expected, as we increase the price of the LARC, the share adopting decreases. If $f(\tilde{\theta}(p)) > 0$, the share adopting the LARC is strictly decreasing in the price of the LARC.

C.2 SDM vs. IDM

Now consider adoption rates and price effects for our two experimental groups: SDM and IDM. Prior to receiving the counseling intervention, it is assumed that the distribution of $\theta's$ is the same in SDM and IDM (by nature of random assignment), i.e., $F^{pre}_{SDM}(\theta) = F^{pre}_{IDM}(\theta)$ where $F^{pre}_{SDM}(\theta)$ ($F^{pre}_{IDM}(\theta)$) denotes the distribution of $\theta's$ for SDM (IDM) individuals prior to receiving counseling. Thus, adoption of the LARC prior to the counseling intervention would be the same in these two groups. However, we are of course interested in adoption of the LARC post-counseling.

For simplicity, assume that IDM does not alter individual uncertainty about whether their return to the LARC is q_l or q_h, i.e., $F^{pre}_{IDM}(\theta) = F^{post}_{IDM}(\theta)$.[3] Conversely, because SDM results in more methods being discussed in greater detail, assume that the SDM intervention reduces individual uncertainty about their type. For simplicity, assume they learn their type with certainty so that $F^{post}_{SDM}(\theta)$ is perfectly bi-modal with a mass point at 0 and a mass point at 1:[4]

$$F^{post}_{SDM}(\theta) = \begin{cases} 1 & \text{if } \theta = 1 \\ 1 - m & \text{if } \theta \in [0, 1) \end{cases}$$

where m denotes the mass at 1 and $1 - m$ denote the mass at 0.

Finally, let $\underline{p}$ denote the lowest price offered and $\overline{p}$ denote the highest price offered. Provided that $\tilde{\theta}(\overline{p}) < 1$ and $\tilde{\theta}(\underline{p}) > 0$ (i.e., some types are willing to adopt the LARC at the high price, and some types are not willing to adopt the LARC at the low price), the share of SDM individuals adopting the LARC (post-counseling) is equal to $m \ \forall \ p \in [\underline{p}, \overline{p}]$ as $1 - F^{post}_{SDM}(\tilde{\theta}(p)) = m \ \forall \ p \in [\underline{p}, \overline{p}]$ (see

[3] This assumption is not necessary. We can easily allow for $F^{pre}_{IDM}(\theta) \neq F^{post}_{IDM}(\theta)$. As will be seen, all we require is that $F^{post}_{IDM}(\theta)$ has a positive density of marginal individuals under the observed prices (i.e., there exist individuals with $\theta's$ close to $\tilde{\theta}(p)$), and that IDM doesn't shift too many individuals' $\theta's$ sufficiently close to 1 such that take-up is higher under IDM relative to SDM.

[4] Note, in order to generate limited price effects for SDM individuals, we do not require that the reduction in uncertainty is sufficiently strong such that the distribution of $\theta's$ is perfectly bi-modal with mass points at the two extremes. This is a simplifying assumption. We just require a strong enough reduction such that individuals who would've been close to marginal under the observed prices (i.e., originally have $\theta's$ close to $\tilde{\theta}(p)$) are no longer close to marginal.

Equation 2). Thus, we will not observe price effects (a reduction in the share adopting the LARC as we move from $\underline{p}$ to $\overline{p}$) in the SDM group as:

$$S_{SDM}(\overline{p}) - S_{SDM}(\underline{p}) = m - m = 0$$

Conversely, provided that some IDM individuals are "marginal", i.e., have $\theta \in [\tilde{\theta}(\underline{p}), \tilde{\theta}(\overline{p})]$, we will observe price effects for the IDM group as $F^{post}_{IDM}(\tilde{\theta}(\overline{p})) > F^{post}_{IDM}(\tilde{\theta}(\underline{p}))$ implying:

$$S_{IDM}(\overline{p}) - S_{IDM}(\underline{p}) = 1 - F^{post}_{IDM}(\tilde{\theta}(\overline{p})) - \left(1 - F^{post}_{IDM}(\tilde{\theta}(\underline{p}))\right) < 0$$

Lastly, provided that enough individuals in the SDM group are truly high types (i.e., enough people learn they have $q = q_h$ post SDM counseling), we will also observe higher adoption of the LARC in the SDM group relative to the IDM group under all prices, i.e., $m > 1 - F^{post}_{IDM}(\tilde{\theta}(p)) \ \forall \ p \in [\underline{p}, \overline{p}]$.[5] It seems reasonable to assume that enough people are truly high types in our experimental sample given that LARCs are recommend first for 88% of individuals in this sample (see Table 6).

[5]Note, using this model to explain why MIM has non-zero price effects but has higher LARC adoption relative to SDM, the following must be true. The number of individuals with "high" $\theta's$ in MIM must be larger than the number of "high" θ individuals in SDM post-intervention: $1 - F_{MIM}(\tilde{\theta}(p)) > 1 - F_{SDM}(\tilde{\theta}(p)) = m$ for $p \in [\underline{p}, \overline{p}]$. This makes sense given individuals wanting a LARC prior to counseling are assigned to the MIM group (i.e., "high" θ individuals are allocated to the MIM group). However, in order to observe price effects in MIM but not SDM, it must be that the reduction in uncertainty in SDM is sufficiently strong such that individuals in SDM are more certain than MIM individuals post-counseling, i.e., there exists some MIM individuals who are marginal under offered prices: i.e., $f_{MIM}(\tilde{\theta}(p)) \neq 0$ for some $p \in [\underline{p}, \overline{p}]$.

D The job-support tool

A multi-disciplinary team of health care providers, public health experts, and economists developed the tablet-based app, which assists nurses conducting contraceptive counseling sessions. Specifically, a working group comprised of experts from the Ministry of Health (Department of Family Health), professors of obstetrics and gynecology from HGOPY, the head FP nurse from HGOPY, and experts in public health, development economics, and behavioral science from the World Bank were part of a working group that developed the app during a week-long workshop in 2018. Once the working group agreed on the broad outlines of the app, a digital consultant was hired by the World Bank to code the app into tablets for use by the nurse counselors. During this phase, the nurses at HGOPY, as well as Prof. Dohbit Sama (one of the co-authors of this study) were involved in the beta testing of the app to iron out bugs and improve the usability of the app during an extended period of back and forth between the developers and the practitioners. Once the app was close to being finalized and the staff at HGOPY were happy with it, it was tested informally by doctors and nurses to ensure that the algorithm worked as expected and that the tablet-based app was a reliable job-support tool for the nurse counselors, rather than causing them additional work.

D.1 What does tablet-based job-support tool do?

The structure of the counselling session as guided by the job-support tool is not fundamentally different than the standard practice –worldwide and at HGOPY. In other words, the tool does not require any new knowledge or training on part of the provider. It simply is a job-aid that allows her to conduct the counseling session more efficiently. The process of family planning counselling using the job-support tool consists of three main sections, as such:

1. Introduction:
   - (a) Welcome the client, explain the purpose of the session (talk about her life and goals, healthy families, pregnancy spacing, safe sex, and contraceptive methods), and clarify that the session is private and confidential.
   - (b) Collect basic demographic information (age, marital status, education, primary activity, religion, and neighbourhood)
   - (c) Discuss client's plans for having children in the future, how long she would like to wait before getting pregnant, how many more children she would like to have, and healthy birth spacing
   - (d) Cover her birth history and establish her breastfeeding status
   - (e) Conduct a pregnancy check
2. Consultation and needs assessment:
   - (a) Discuss current method of birth control used by the client, if any. Discuss her experience with the method, how long she has been using it, and assess whether she would like to continue or switch

(b) Discuss any methods that she might be worried about. Any methods she has in mind that she is curious about.
(c) Clarify any questions or misconceptions the client might have about any contraceptive methods
(d) Ask her about her preferences regarding side effects concerning:
    i. Increased bleeding and cramping,
    ii. Decreased bleeding, spotting, and amenorrhea, and
    iii. Weight gain
(e) Obtain her medical history to avoid the adoption of contra-indicated methods. Take blood pressure and measure the height and weight of the client.

3. Method choice and follow-up:

    (a) Depending on the intervention condition, either ask the client to choose the modern method she would like to discuss first OR ask her whether she would like to discuss the method that is recommended by the tool as being the most suitable for her needs. When the client makes her choice as to which method she would like to discuss, the provider presents neutral, evidence-based, and understandable information on the effectiveness and the side effects of that method with the help of printed and laminated cue cards (see Appendix section D.4 for an example cue card).
    (b) Answer client's questions and concerns about the method being discussed. Listen to the client carefully and counsel her individually, based on her needs assessment.
    (c) Ask the client whether she would like to adopt the method or discuss another method. Discuss next preferred method, and so on, until the client decides to adopt or leave with no method.
    (d) Adoption of chosen method, as appropriate, along with documentation of consent for adopting a modern method, such as the pill, injectable, implant, or IUD.
    (e) Provide information on method use and follow-up mechanisms for switching or discontinuing selected method.

4. Conclusion:

    (a) Discuss the importance of dual protection from sexually transmitted diseases and provide the client with a package of condoms.
    (b) Schedule the next appointment, as appropriate.
    (c) Provide the client information about the study and seek her informed consent to participate in the study.

D.2 Medical eligibility and contra-indications

We use the U.S. Centre for Disease Control and Prevention's recommendations for "U.S. Medical Eligibility Criteria for Contraceptive Use, 2016" to determine methods that are contraindicated for various conditions the clients may have. The main considerations are the following:

- Recent delivery of a baby
- Breastfeeding
- Unexplained vaginal bleeding
- Current blood pressure/history of hypertension
- Risk factors such as older age (>35), smoking, diabetes
- Medications such as TB drugs, Barbiturates, and Antiretroviral drugs

A large number of medical eligibility rules relating to the conditions above are programmed into the algorithm and when a condition is satisfied, the method is ruled out and excluded from rankings. When this happens, the job-support tool displays a message at the top of the method choice section that certain methods are being excluded due to medical eligibility criteria, so that the provider can explain the client why she is not being given the option to discuss that method.

D.3 Method rankings

In order to produce the method ranking, the tablet-based job-support tool takes into account the clients' preferences regarding how long they would like to wait before becoming pregnant and the importance they attach to avoiding several common various side effects.

There are three key criteria that are used by the algorithm:

1. How long they would like to wait before becoming pregnant.
2. How strongly they feels about avoiding the following three categories of side effects:
   (a) Increased bleeding and cramping,
   (b) Decreased bleeding, spotting, and amenorrhea, and
   (c) Weight gain.
3. Typical use effectiveness of each method.

The algorithm generates a score for each method based on these criteria. The scoring mechanism uses evidence on the average side effects of each method from the existing peer-reviewed literature. The typical use of effectiveness data underlying the third criteria is based off of from peer-reviewed literature (namely Trussell and Guthrie 2011).

For example, if the client feels strongly about minimizing the chances of all three categories of side effects and would like to wait more than one year before getting pregnant, the ranking of methods (from most suitable to least suitable) is as follows: IUD, pill, (lactational amenorrhea method or

LAM), implant, and the injectable.[6] Note that in this example, the pill, which is a short-acting method with a typical use effectiveness much lower than the implant and the injectable, is ranked above both methods because of the client's preferences regarding side effects.

In contrast, consider a client who wishes to have no more children and does not care about any of the side effects. The method rankings for such an individual is: IUD, implant, injectable=(LAM), and the pill. The reader will now notice that because the client is interested in avoiding pregnancies altogether and is not concerned with side effects, long-acting methods are ranked higher, while the pill, which has the mildest expected side effects, is ranked at the bottom. The algorithm sometimes produces identical scores for two or more methods that result in a tie in the rankings. In such cases, the client is told that two (or more) methods are equally suitable for her and the tablet uses an internal random number generator to decide the ordering of the tied methods for discussion.

Table 6 below presents method rankings for 24 possible cases of side effects preferences and desired method duration (or, more accurately, the duration the client would like to wait before becoming pregnant). Including MiM clients, from whom side effects preferences were not elicited, and various adjustments and tie-breakers for duration and the client being a nulliparous adolescent bring the possible number of rankings to 69, a full table of which can be made available by request.

Importance of side-effects in counselling: During the trial, duration, effectiveness, and convenience were the top reasons mentioned by clients with a method in mind. In contrast, barely anyone mentioned side effects (or lack thereof) as the reason they were seeking to adopt a method. This is not surprising because side effects for the user are not revealed until after adoption, which can cause the user to consider switching or discontinuing their method. Hubacher et al. (2017) show, in the U.S. context, that the most important reason for discontinuations is side effects, especially for LARCs. Formative qualitative work in preparation for this trial revealed that a lot of people, both providers and clients, are confused about the idiosyncratic nature of the side effects of contraceptives. Providers also struggled to discuss these with clients. Hence, we decided to include detailed questions on side effects preferences to (a) aid the providers in explaining them to the clients and (b) minimize discontinuations by ranking methods according to the strength of these preferences. Potential users of the app can revise the rankings as they wish by including more or less attributes (and eliciting preferences on those from clients), as well as changing the scoring and/or the weighting scheme.

The exact script of the english version of the app is included below. It is important to note that these questions are used by the nurse counsellors as entry points for a broader discussion about possible side-effects and contraception, in which the various available methods are often touched upon, but during which the focus is always to elicit the clients' preferences regarding their experience with contraception. During this phase the clients also often ask many questions and clarifications, such that these three questions constitute a significant portion of the counselling session.

1. *Increased bleeding:* "Some methods can cause increased menstrual bleeding and cramping, though this effect subsides for most women after the first three months. How much will it

[6] The LAM method is included in the rankings if a client has (i) given birth in the past six months; (ii) is fully breastfeeding; and (iii) has not menstruated since birth; and excluded otherwise.

bother you if you experience increased cramping or bleeding during the first three months?"

2. *Decreased bleeding:* "Some methods cause decreased menstrual bleeding over time with some women eventually not having a period at all. Some methods can cause spotting. Absence of bleeding is definitely NOT harmful to your health. Nor is spotting. In fact, some women consider absence of bleeding to be convenient and it is an added health benefit. How much will it bother you if you experience spotting or amenorrhea?"

3. *Weight gain:* "A majority of women do not experience any weight gain using the methods we will discuss. However, a minority of women can experience significant weight gain with some of the methods. If this happens to you, we can help you manage this issue. How much will it bother you if you experience significant weight gain?"

Table S10: Method rankings.

Side effects			Spacing	Method ranking				
Bleeding increase	Bleeding decrease	Weight gain	Time until next preg.	No. 1	No. 2	No. 3	No. 4	No. 5
			≥1 year	IUD	Pill	LAM	Implant	Injectable
Yes	Yes	Yes	<1 year	IUD/Pill	IUD/Pill	LAM	Implant	Injectable
			No more	IUD	Pill	LAM	Implant	Injectable
			≥1 year	Implant/IUD	Implant/IUD	Pill	LAM	Injectable
Yes	Yes	No	<1 year	Implant	IUD/Pill	IUD/Pill	LAM	Injectable
			No more	Implant	IUD	Pill	LAM	Injectable
			≥1 year	LAM	IUD/Pill	IUD/Pill	Implant	Injectable
Yes	No	Yes	<1 year	LAM	Pill	IUD/Implant	IUD/Implant	Injectable
			No more	LAM	Pill	Implant	IUD	Injectable
			≥1 year	Implant	LAM	IUD/Pill	IUD/Pill	Injectable
Yes	No	No	<1 year	Implant	LAM	Pill	IUD	Injectable
			No more	Implant	LAM	Pill	IUD	Injectable
			≥1 year	IUD	Implant/Pill	Implant/Pill	LAM	Injectable
No	Yes	Yes	<1 year	IUD	Implant/Pill	Implant/Pill	LAM	Injectable
			No more	IUD	Implant	Pill	LAM	Injectable
			≥1 year	IUD	Implant	Pill	Injectable/LAM	Injectable/LAM
No	Yes	No	<1 year	Implant/IUD	Implant/IUD	Pill	LAM	Injectable
			No more	Implant	IUD	Pill	LAM	Injectable
			≥1 year	IUD	LAM	Implant/Pill	Implant/Pill	Injectable
No	No	Yes	<1 year	IUD	LAM	Implant/Pill	Implant/Pill	Injectable
			No more	IUD	LAM	Implant	Pill	Injectable
			≥1 year	Implant/IUD	Implant/IUD	Injectable/LAM	Injectable/LAM	Pill
No	No	No	<1 year	Implant	IUD	LAM	Pill	Injectable
			No more	Implant	IUD	LAM	Pill	Injectable

Notes: This table shows the possible method rankings and how they depend on the client's preferences with respect to side-effects and the spacing they hope to achieve until their next pregnancy. In the first three columns, a 'Yes' indicates that the client would find experiencing the relevant side effect bothersome. Ties are broken randomly, except for in cases where the client is a nulliparous adolescent, in which ties are broken against the IUD.

D.4 Consultation cue cards

This section shows the two sides of the consultation cue cards for the implant:

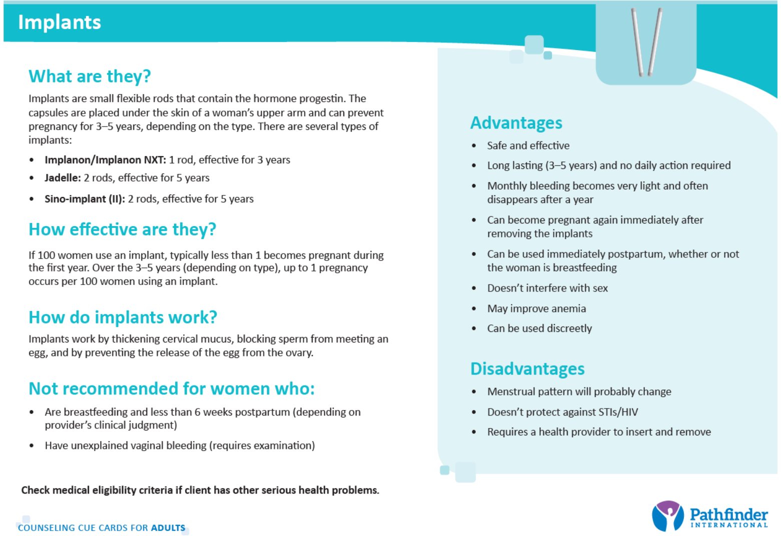

Implants

What are they?

Implants are small flexible rods that contain the hormone progestin. The capsules are placed under the skin of a woman's upper arm and can prevent pregnancy for 3–5 years, depending on the type. There are several types of implants:

- **Implanon/Implanon NXT:** 1 rod, effective for 3 years
- **Jadelle:** 2 rods, effective for 5 years
- **Sino-implant (II):** 2 rods, effective for 5 years

How effective are they?

If 100 women use an implant, typically less than 1 becomes pregnant during the first year. Over the 3–5 years (depending on type), up to 1 pregnancy occurs per 100 women using an implant.

How do implants work?

Implants work by thickening cervical mucus, blocking sperm from meeting an egg, and by preventing the release of the egg from the ovary.

Not recommended for women who:

- Are breastfeeding and less than 6 weeks postpartum (depending on provider's clinical judgment)
- Have unexplained vaginal bleeding (requires examination)

Advantages

- Safe and effective
- Long lasting (3–5 years) and no daily action required
- Monthly bleeding becomes very light and often disappears after a year
- Can become pregnant again immediately after removing the implants
- Can be used immediately postpartum, whether or not the woman is breastfeeding
- Doesn't interfere with sex
- May improve anemia
- Can be used discreetly

Disadvantages

- Menstrual pattern will probably change
- Doesn't protect against STIs/HIV
- Requires a health provider to insert and remove

Check medical eligibility criteria if client has other serious health problems.

COUNSELING CUE CARDS FOR **ADULTS**

Pathfinder INTERNATIONAL

Figure S8: Example cue card used during the consultation, Implant (Front of the card)

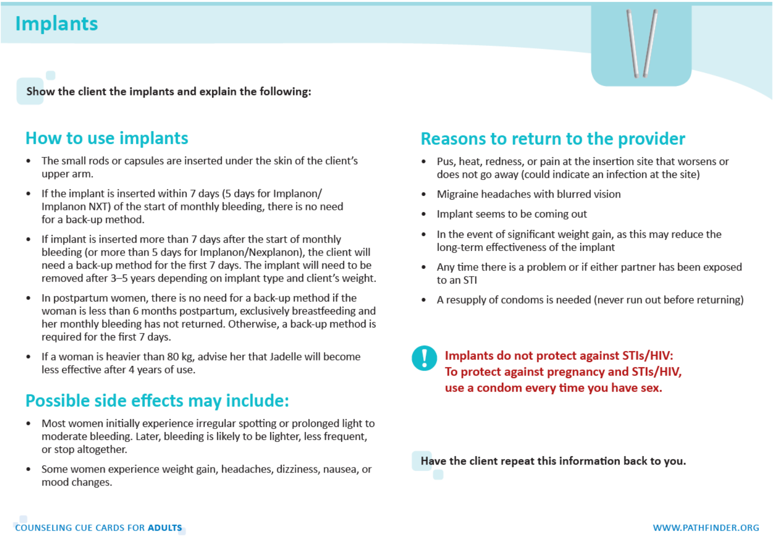

Implants

Show the client the implants and explain the following:

How to use implants

- The small rods or capsules are inserted under the skin of the client's upper arm.
- If the implant is inserted within 7 days (5 days for Implanon/Implanon NXT) of the start of monthly bleeding, there is no need for a back-up method.
- If implant is inserted more than 7 days after the start of monthly bleeding (or more than 5 days for Implanon/Nexplanon), the client will need a back-up method for the first 7 days. The implant will need to be removed after 3–5 years depending on implant type and client's weight.
- In postpartum women, there is no need for a back-up method if the woman is less than 6 months postpartum, exclusively breastfeeding and her monthly bleeding has not returned. Otherwise, a back-up method is required for the first 7 days.
- If a woman is heavier than 80 kg, advise her that Jadelle will become less effective after 4 years of use.

Possible side effects may include:

- Most women initially experience irregular spotting or prolonged light to moderate bleeding. Later, bleeding is likely to be lighter, less frequent, or stop altogether.
- Some women experience weight gain, headaches, dizziness, nausea, or mood changes.

Reasons to return to the provider

- Pus, heat, redness, or pain at the insertion site that worsens or does not go away (could indicate an infection at the site)
- Migraine headaches with blurred vision
- Implant seems to be coming out
- In the event of significant weight gain, as this may reduce the long-term effectiveness of the implant
- Any time there is a problem or if either partner has been exposed to an STI
- A resupply of condoms is needed (never run out before returning)

Implants do not protect against STIs/HIV: To protect against pregnancy and STIs/HIV, use a condom every time you have sex.

Have the client repeat this information back to you.

COUNSELING CUE CARDS FOR **ADULTS**

WWW.PATHFINDER.ORG

Figure S9: Example cue card used during the consultation, Implant (Back of the card)
